# Concentrations of criteria pollutants in the contiguous U.S., 1979 – 2015: Role of prediction model parsimony in integrated empirical geographic regression

**DOI:** 10.1371/journal.pone.0228535

**Published:** 2020-02-18

**Authors:** Sun-Young Kim, Matthew Bechle, Steve Hankey, Lianne Sheppard, Adam A. Szpiro, Julian D. Marshall

**Affiliations:** 1 Department of Cancer Control and Population Health, Graduate School of Cancer Science and Policy, National Cancer Center, Goyang-si, Gyeonggi-do, Korea; 2 Department of Environmental and Occupational Health Sciences, University of Washington, Seattle, WA, United States of America; 3 Department of Civil and Environmental Engineering, University of Washington, Seattle, WA, United States of America; 4 School of Public and International Affairs, Virginia Polytechnic Institute and State University, Blacksburg, VA, United States of America; 5 Department of Biostatistics, University of Washington, Seattle, WA, United States of America; Lancaster University, UNITED KINGDOM

## Abstract

National-scale empirical models for air pollution can include hundreds of geographic variables. The impact of model parsimony (i.e., how model performance differs for a large versus small number of covariates) has not been systematically explored. We aim to (1) build annual-average integrated empirical geographic (IEG) regression models for the contiguous U.S. for six criteria pollutants during 1979–2015; (2) explore systematically the impact on model performance of the number of variables selected for inclusion in a model; and (3) provide publicly available model predictions. We compute annual-average concentrations from regulatory monitoring data for PM_10_, PM_2.5_, NO_2_, SO_2_, CO, and ozone at all monitoring sites for 1979–2015. We also use ~350 geographic characteristics at each location including measures of traffic, land use, land cover, and satellite-based estimates of air pollution. We then develop IEG models, employing universal kriging and summary factors estimated by partial least squares (PLS) of geographic variables. For all pollutants and years, we compare three approaches for choosing variables to include in the PLS model: (1) no variables, (2) a limited number of variables selected from the full set by forward selection, and (3) all variables. We evaluate model performance using 10-fold cross-validation (CV) using conventional and spatially-clustered test data. Models using 3 to 30 variables selected from the full set generally have the best performance across all pollutants and years (median R^2^ conventional [clustered] CV: 0.66 [0.47]) compared to models with no (0.37 [0]) or all variables (0.64 [0.27]). Concentration estimates for all Census Blocks reveal generally decreasing concentrations over several decades with local heterogeneity. Our findings suggest that national prediction models can be built by empirically selecting only a small number of important variables to provide robust concentration estimates. Model estimates are freely available online.

## Introduction

Regulatory monitors and project-based monitoring campaigns typically provide air pollution measurements that are limited in space and time. Empirical models are a cost-effective approach to estimate fine-scale exposures to air pollution. Recent population-level studies of air pollution have relied on empirical models to estimate long-term concentrations of outdoor air pollution based largely on observation-driven geostatistical approaches [1–3] or hybrid approaches that incorporate satellite-based observations of air quality and theory-based mechanistic models with geostatistical approaches [4–7]. These model predictions are used to assess population-level characteristics of air pollution, such as health effects [8–11], the burden of disease [12,13], and exposure disparities [14,15].

Many empirical models of air pollution are developed using a large suite of input data often including hundreds of geographic covariates (e.g., traffic, population, land use) with the goal of predicting concentrations at locations lacking monitoring data [16]. More recently, studies have included estimates of air pollution from mechanistic models [17,18] and satellite-based air pollution measurements such as tropospheric nitrogen dioxide (NO_2_) column abundance and Aerosol Optical Depth (AOD) [19,20]. These regional air pollution estimates are particularly useful for national- or global-scale prediction where air pollution measurements are sparse over large areas [4,7,21–25]. To incorporate and prioritize information from the many hundreds of predictor variables, studies typically employ conventional statistical techniques such as variable selection, shrinkage, and dimensional-reduction [1,26,27]; more recently some studies have applied machine learning techniques such as neural network [4,28].

Computational demands of applying models that include hundreds of variables are large, especially for the models aiming for extended prediction areas such as national scale. Yet, there is little guidance in the literature regarding the added benefit of using hundreds of variables versus parsimonious models based on a few or a couple dozen empirically-selected variables. Furthermore, although some national-scale models exist for specific years and pollutants for particulate matter less than or equal to 2.5 or 10 microns in diameter (PM_2.5_ or PM_10_), NO_2_, or ozone [25,27,29], empirical models developed under a unified framework do not currently exist for most criteria pollutants across all years with regulatory monitoring data in the U.S. This article aims to address both of those gaps. Specifically, we develop, test, and compare full versus parsimonious national models that predict annual average concentrations of six criteria pollutants and for all years with available monitoring data during 1979–2015. We test the hypothesis that model performance is better with more variables than with a smaller number of empirically selected variables from the full dataset. We compare the performance from the models using different numbers of variables within an identical modeling framework to focus on the comparison across different sizes of subsets. Then, we select the best-performing models to generate concentration estimates for all residential Census Block centroids in the contiguous U.S. for all modeling years with the goal of making our model predictions available freely online.

We refer to our models as “Integrated Empirical Geographic” (IEG) regression models to indicate key characteristics of the model and to be effectively acknowledgeable in other planned analyses: “integrated” because they include many datasets (land use, satellite-derived measures of air pollution, and emission estimates); “empirical” because the relationship derived is empirical (rather than based on theory [physics, chemistry]) and because the model is based on measured concentrations; and, “geographic” because the model is based on geography and geographic variables, and also because it includes kriging, a geostatistical method for spatial prediction.

## Materials and methods

### Regulatory monitoring data for criteria pollutants

We downloaded measurements of six criteria pollutants including PM_10_, PM_2.5_, NO_2_, sulfur dioxide (SO_2_), carbon monoxide (CO), and ozone (O_3_) at all Air Quality System (AQS) monitoring sites for all available years from 1979 through 2015 via the U.S. Environmental Protection Agency (EPA) AQS data repository (https://www.epa.gov/outdoor-air-quality-data) (S1 Fig). Criteria pollutants are a list of air pollutants that are known as their harmful effects on health, and monitored and managed on a national level to achieve the compliance with the air quality standards. Whereas gaseous pollutants such as NO_2_, SO_2_, CO, and O_3_ are measured every hour, PM is collected on the daily basis. NO_2_, SO_2_, and ozone are available for the entire period (1979–2015); CO, PM_10_, and PM_2.5_ are available starting in 1990, 1988, and 1999, respectively. For PM_10_ and PM_2.5_, we used data from the Federal Reference Method (FRM) and Integrated Monitoring of Protected Visual Environments (IMProVE) networks (http://vista.cira.colostate.edu/Improve/).

We computed annual averages for all pollutants (except ozone) at sites that meet our inclusion criteria, as follows. We computed 24-hour averages for monitors with 18 or more valid hourly measurements in that day, and then computed annual averages at sites with a minimum number of operating days (244 days for daily/hourly measurements, 61 days for 1-in-3 day measurements, and 41 days for 1-in-6 day measurements) during a year and no more than 45 consecutive days without a measurement. For ozone, we computed the daily maximum of the 8-hour moving average from hourly measurements for monitors with 18 or more operating hours during the day and computed an ozone season average from May through September. We selected these summer-season averages of daily 8-hour maximum for ozone because ozone production is predominant in summer through photochemical reaction catalyzed by heat and sunlight and it is likely that its health effect is mostly affected by summer time ozone concentrations. The IEG regression modeling was done after applying square root transformation to all pollutant concentrations to meet the normality assumption.

### Geographic variables

We considered > 900 geographic variables as independent variables for our IEG models, in eleven categories: traffic, population, urban land-use or land-cover, rural land-use or land-cover, elevation, vegetation, imperviousness, industrial emissions, position, source, and satellite air pollution estimates (S1 Table). S2 Fig shows the diagram of the procedures including data preprocessing and variable computation (detailed information is also available in https://www.uwchscc.org/MESAAP/Documents/MESAAirDOOP.pdf). To reflect changes of land use characteristics over time, we obtained the two types of land use variables from ground-based datasets generated in 1970s and 1980s, and satellite and aerial imagery in 2006. The variables were computed as summaries within buffer areas between 50 meters and 15 kilometers (0.05, 0.1, 0.15, 0.3, 0.4, 0.5, 0.75, 1, 1.5, 3, 5, 10, and 15 km) that were applied differently by the variables depending on their local or regional impacts on air pollution (S1 Table). From > 900 variables, we excluded variables with little spatial variability (e.g., same values at the 10^th^ and 90^th^ percentiles) or few unique values. This exclusion resulted in reduction of the number of variables to an average of ~350 as the full set of variables for a given pollutant and year (S3 Fig).

Traffic variables are distance to the nearest road and sum of road lengths within eleven circular buffers (0.05, 0.1, 0.15, 0.3, 0.4, 0.5, 0.75, 1, 1.5, 3, and 5 km) based on TeleAtlas data (http://www.teleatlas.com/OurProducts/MapData/Dynamap/index.htm). Population variables are the number of people in eight circular buffers (0.5, 0.75, 1, 1.5, 3, 5, 10, and 15 km), based on year-2000 U.S. Census population (http://arcdata.esri.com/data/tiger2000/tiger_download.cfm). Land use variables in 1970s and 1980s are percent of areas in circular buffers for various land use characteristics such as residential, industrial, commercial, and agricultural land use identified by the U.S. Geological Survey (http://water.usgs.gov/GIS/dsdl/ds240/index.html). Land cover variables based on satellite imagery in 2006 are percent of areas in circular buffers for land use characteristics such as developed high and low density obtained from the Multi-Resolution Land Cover Characteristics (MRLC) Consortium (http://www.mrlc.gov/index.php). Elevation is the absolute elevation measurement at a given location and relative elevation compared to elevation at grid points in a circular buffer area, calculated from national elevation dataset based on satellite imagery (http://nationalmap.gov/elevation.htm). Vegetation variables are normalized difference vegetation index computed from satellite imagery (http://glcf.umd.edu/data/ndvi/) computed in circular buffer areas. Emission variables are the total amount emission estimates in circular buffer areas based on national emission inventory data (http://www.epa.gov/ttn/chief/net/2002inventory.html).

We obtained and computed annual satellite-based estimates of air pollution concentrations for PM_2.5_, NO_2_, SO_2_, CO, and formaldehyde (HCHO) (S2 Table); see the Supporting Information for details on the specific steps. The net result is satellite-derived annual-average estimates for PM_2.5_ (1998–2014; 0.1° × 0.1° grid) [7], NO_2_ (2004–2015; 0.1° × 0.1° grid) [30], SO_2_ (2005–2016; 0.25° × 0.25° grid) [31,32], and CO (2001–2016; 0.25° × 0.25° grid) [33], and a multiyear average for HCHO (2005–2016; 0.25° × 0.25° grid) [34]. We used the estimate on the grid where the target location is located.

### Modeling approach

Our approach builds on a universal kriging framework, as described in our previous studies [25,27,35], that partitions annual average concentrations into two components [36]: variance and mean. The variance component is modeled with variogram using exponential covariance function and three covariance parameters: range (the distance at which spatial correlation exists), partial sill (spatial variability), and nugget (non-spatial variability) (see the S1 File). The mean component includes a few dimension-reduced summary predictors estimated using partial least squares (PLS) from the geographic variables offered. We estimated two and three PLS predictors from conventional and clustered cross-validation, respectively, (see the next section, “Model Evaluation”) based on the previous study that showed the best model performance using two to three PLS predictors in the same modeling approach for the NO_2_ national model in U.S. [25]. The mean component is equivalent to the linear regression model often referred to as land use regression (LUR) with PLS data-reduction. Whereas other dimension reduction approaches such as principal component analysis solely rely on correlation of covariates, PLS predictors are estimated based on the correlation between covariates and the outcome; PLS was adopted in several previous prediction models [2,3,18,25,27,35]. The summary predictors not only incorporate various geographic characteristics, they also avoid producing extreme predictions [37]. All regression parameters of PLS predictors and covariance parameters were estimated by maximum likelihood method. The model was applied by each pollutant and each year. We implemented all IEG models in R (ver. 3.5.1; R Development Core Team, Vienna, Austria, https://www.r-project.org/).

To investigate the role of model parsimony, we empirically selected via forward selection a specific number of variables from the full set of ~ 350 to offer the PLS; we investigated how model performance varies depending on the number of variables selected. The number of variables selected ranges from zero (i.e., no variables–this is ordinary kriging that assumes a constant mean without any variables) to the full covariate database, with several intermediate values up to approximately one third of the all variables (3-, 5-, 7-, 10-, 13-, 16-, 20-, 25-, 30-, 60-, 90-, and 120-variable models). For example, the 20-variable model would involve forward selection to select the best 20 variables from the full set, followed by PLS data-reduction to identify two or three PLS components comprised of those 20 variables, and regression modeling based on those two or three PLS components. PLS has been shown to be a useful tool for variable selection in previous studies where the set of variables being considered is limited and there is a clear understanding of their scientific importance [38]. However, we restricted our application of PLS to estimation of summary predictors from selected subsets of variables because we had no a priori reason to limit the full set of ~350 variables.

We hypothesized that adding more variables will always improve the model somewhat, but perhaps by diminishing amounts as more variables are added. In that case, there may be a “point of diminishing returns”: a number of variables for which adding more variables yields little additional benefit.

### Model evaluation

We evaluated models using two types of 10-fold cross-validation (CV): conventional and spatially clustered [25]. Whereas conventional CV randomly generates groups of sites as training and test data sets, spatially clustered CV is based on those groups constructed as specific spatial clusters. For conventional CV, we randomly divided all monitoring sites into 10 groups. Then, we selected one group as the hold-out sites, developed models using the remaining data, and predicted air pollution concentrations at hold-out sites. This process was repeated separately for each of the 10 groups to create a pseudo-independent test data set. Spatially clustered CV is similar except that the 10 groups are spatial clusters identified using k-means clustering (S4 Fig) [25]. Conventional CV reflects model performance at a random location, whereas clustered CV reflects model performance far from a monitor. For dense monitoring networks, such as PM_2.5_ in the U.S., conventional CV may be more representative of model performance where most people live.

CV statistics include root-mean-square error (RMSE) and the MSE-based R-squared statistic (R^2^). The MSE-based R^2^ is calculated as 1 minus the ratio of MSE to data variability, whereas a conventional R^2^ is calculated as the squared correlation coefficient. Conventional R^2^ assesses agreement between predictions and observations about the regression line; MSE-based R^2^ instead assesses agreement about the 1:1 line [2, 37]. To allow for comparison across different pollutants, we also computed standardized RMSE (i.e., RMSE divided by the mean concentration across all sites). For each pollutant and year, the “best” and “worst” models are identified based on R^2^ and standardized RMSE from both conventional and clustered CV.

### Sensitivity analyses

As described next, we conducted sensitivity analyses to investigate the contribution of a specific category of geographic variables, the impact of model evaluation choices, the performance of an alternative modeling approach, and the impact of different pollutant metrics.

To investigate whether a specific category of geographic variables gives higher contribution than others to prediction, we developed the model by separately excluding each category of variables such as traffic, land use, and satellite air pollution estimates (see above and S1 Table) in turn. Then, we investigated which category shows the largest decline in model performance when excluded.

To shed light on whether the selected best and worst models are sensitive to the type of CV approach, we used the best models chosen by one CV and recomputed CV statistics by the other CV (i.e., re-compute conventional CV for the best models chosen by clustered CV, and re-compute clustered CV for the best models chosen by conventional CV). To assess the impact of forward selection during model-building, we replaced forward selection with random selection of the same number of variables and compared model performance. To more completely evaluate our models by addressing model selection as well as estimation of regression and covariance parameters in universal kriging, we constructed our CV to include forward selection and estimation of PLS predictors in addition to parameter estimation as a more complete model evaluation. In addition, we performed an additional CV in which we take out all sites within a certain radius of a buffer instead of one site in conventional CV [39]. This conventional buffer-out CV intends to avoid possible overestimation of model performance resulting from monitoring data collected at the neighboring sites and correlated with those at hold-out sites. For buffer size, we used 50, 100, 200, and 300 km radii based on our investigation of histogram and variogram for monitoring data. As an alternative modeling approach, we applied least absolute shrinkage and selection operator (lasso) [40] to select subsets of variables for PLS and compared the performance to that of our original approach using forward selection. We applied the sensitivity analyses for categories of geographic variables, model evaluation, and modeling approaches to limited examples: two pollutants for NO_2_ and PM_2.5_, and one year in 2000.

Lastly, we tested the robustness of ozone models to other ozone averaging approaches: annual and summer season (May-September) summaries of ozone using 24-hour means, 8-hour means, and 1-hour maximum.

### Prediction

Using the best models for each pollutant and year, we predicted annual average concentrations for the ~7 million residential Census Block centroids in the contiguous U.S. with nonzero population. Then, we computed population-weighted averages at various geographic scales (Census Block Groups, Census Tracts, Counties, States, and contiguous U.S.) based on 2010 Census boundaries, and explored the national distribution of pollutant concentrations over space and time.

## Results

### Summary of monitoring data

**Fig 1:**
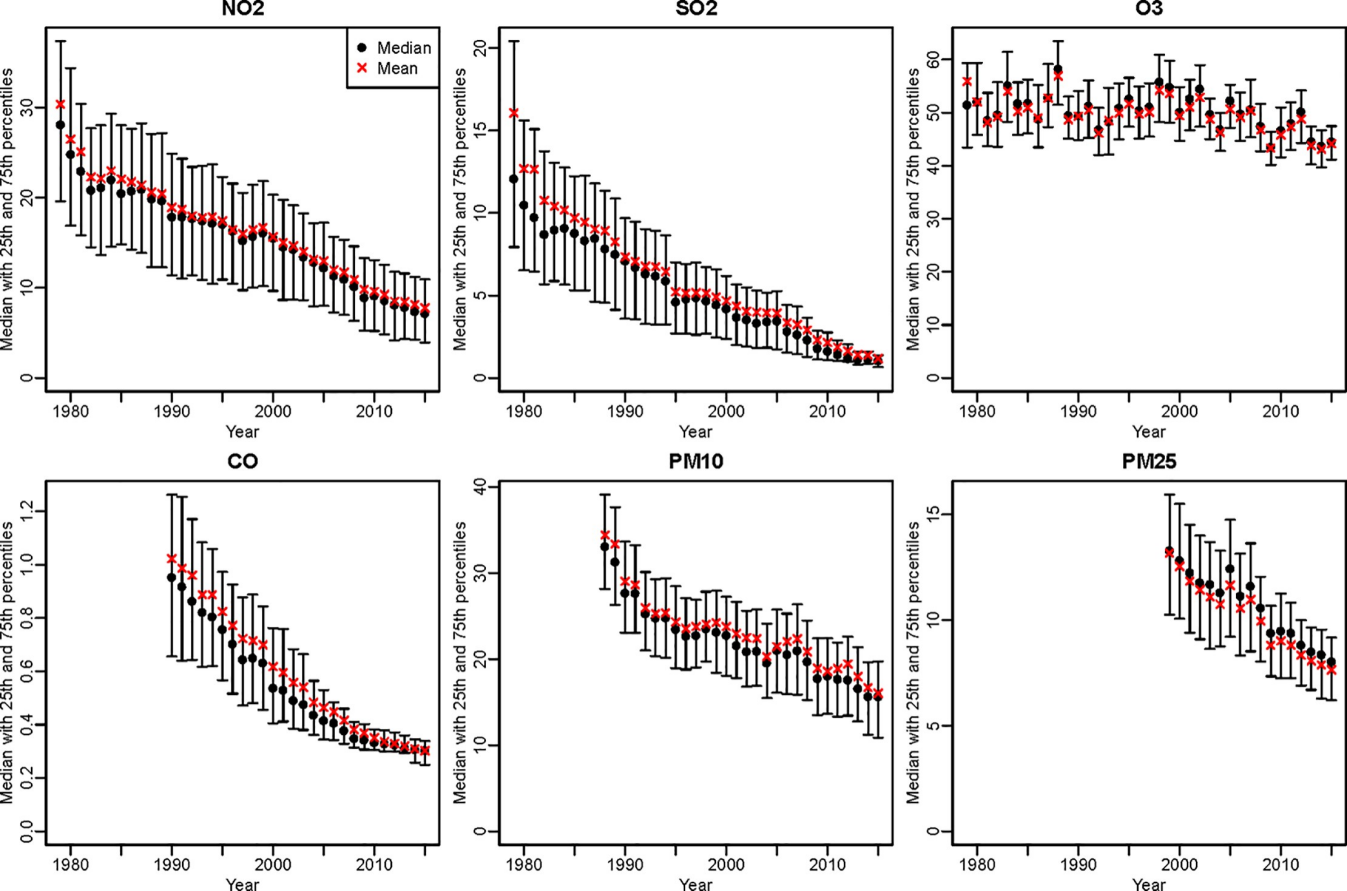
Quantile-based plots of annual average concentrations of six criteria air pollutants across all regulatory monitoring sites for 1979–2015 in the contiguous U.S.

Means and standard deviations of annual average concentrations at AQS monitoring sites decrease over time for all pollutants (S3 Table, Fig 1). During 1980 to 2010, average concentrations decrease almost 6-fold for SO_2_ (from 12.7 to 2.2 ppb) but only 14% for ozone (from 52.0 to 45.8 ppb). For ozone, the 10^th^ percentile concentration decreases less than 2% over 30 years (from 7.8 to 37.2 ppb). From 2000 to 2010, reductions for PM_2.5_ and PM_10_ are 39% and 28%, respectively.

### IEG model performance by number of variables

**Fig 2:**
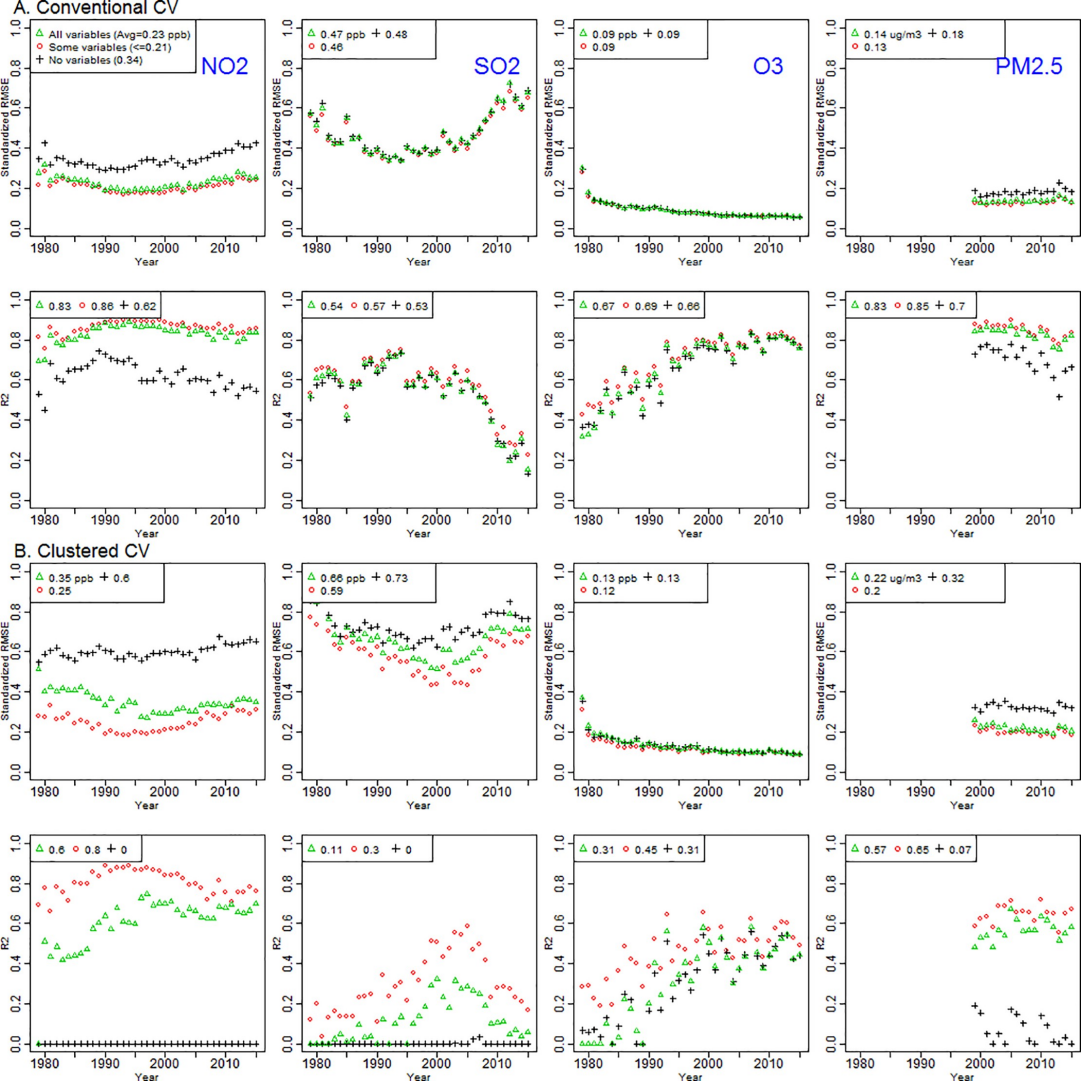
Standardized RMSEs and R^2^s of the national Integrated Empirical Geographic (IEG) models including no, some, and all variables from conventional and clustered cross-validation (CV) during 1979–2015 for the contiguous U.S. by NO_2_, SO_2_, ozone, and PM_2.5_ (triangle: all variables, circle: Some variables (3–30), and cross: no variables; terminology here is the same as in Table 1;).

Different from our hypothesis, adding more variables does not consistently improve model performance, especially for clustered CV (S5 Fig). For all pollutants and for both CV approaches, models using 3–30 variables generally show higher R^2^ and lower standardized RMSE than models using no or all variables (Table 1, Fig 2 and S6 Fig).

**Table 1:** Cross-validation (CV) statistics for the Integrated Empirical Geographic (IEG) regression models by pollutant, year, and numbers of geographic variables (zero variables / between 3 and 30 variables / all variables).

		Conventional CV						Clustered CV					
		Standardized RMSE^a^			R^2^			Standardized RMSE			R^2^		
		0	3–30	All	0	3–30	All	0	3–30	All	0	3–30	All
Pollutant	Year												
NO_2_	2000	0.33^b^	0.19	0.20	0.61	0.87	0.85	0.60	0.23	0.29	0.00	0.82	0.70
(ppb)	2010	0.39	0.23	0.25	0.56	0.84	0.81	0.64	0.33	0.33	0.00	0.68	0.68
SO_2_	2000	0.39	0.38	0.39	0.60	0.63	0.61	0.62	0.47	0.51	0.00	0.44	0.32
(ppb)	2010	0.64	0.63	0.65	0.29	0.31	0.28	0.79	0.65	0.72	0.00	0.26	0.10
O_3_	2000	0.07	0.07	0.07	0.76	0.78	0.78	0.11	0.10	0.11	0.45	0.55	0.51
(ppb)	2010	0.06	0.06	0.06	0.81	0.82	0.81	0.11	0.10	0.11	0.44	0.51	0.44
CO	2000	0.37	0.32	0.34	0.33	0.50	0.43	0.47	0.35	0.43	0.00	0.42	0.12
(ppm)	2010	0.25	0.23	0.25	0.17	0.28	0.20	0.28	0.24	0.28	0.00	0.23	0.00
PM_10_	2000	0.31	0.27	0.28	0.50	0.60	0.59	0.45	0.37	0.39	0.00	0.27	0.20
(μg/m^3^)	2010	0.34	0.29	0.30	0.41	0.57	0.56	0.47	0.37	0.39	0.00	0.33	0.26
PM_25_	2000	0.16	0.12	0.13	0.77	0.86	0.85	0.30	0.21	0.22	0.15	0.59	0.53
(μg/m^3^)	2010	0.17	0.13	0.13	0.73	0.85	0.84	0.31	0.19	0.20	0.14	0.70	0.64

^a^Standardized RMSE is the root mean square error (RMSE) divided by average concentration.

^b^All values of CV statistics are shown by the three levels of selected numbers of variables: for models with zero variables (i.e., ordinary kriging), denoted with “0”; the median among the models with between 3 and 30 variables (3, 5, 7, 10, 13, 16, 20, 24, and 30), denoted “3–30”; and for full models with all variables, denoted “all”.

The no-variable (i.e., ordinary kriging) models generally perform worst (S7 Fig). Selecting best-performing models generally is consistent among metrics (MSE-R^2^, standardized RMSE), and is typically robust to using the two types of CV (S8 Fig). S4 Table shows the medians of three estimated covariance parameters across 10 CV groups and the contribution of PLS predictors to CV-ed predictions by the models including different numbers of variables for NO_2_ and PM_2.5_ in 2000. The contribution of PLS predictors to CV-ed predictions was computed as the proportion of CV-ed predictions by PLS predictors to CV-ed predictions by PLS predictors and kriging. For NO_2_, median covariance parameters are similar between some and all variable models but notably different from those in no-variable models particularly for the range parameter. However, parameters for PM_2.5_ were similar between some variable models compared to no or all variable models. Overall, the contribution of PLS predictors to CV-ed predictions is dominant with small contributions of spatial variability as shown in small partial sill. These patterns were consistent between some-variable and all-variable models for both pollutants.

### IEG model performance by CV

The patterns of good model performance with small numbers of variables are generally similar between the two CV approaches. However, CV results consistently indicate better model performance using conventional CV than using clustered CV (Table 1, Fig 2 and S6 Fig), reflecting poor performance when there are no monitors in the vicinity. Considering all pollutants and years, median R^2^ and standardized RMSE, based on conventional CV, for the best models are 0.66 (interquartile range [IQR]: 0.57–0.83) and 0.23 (0.13–0.31), respectively. Analogous values for clustered CV are median R^2^ of 0.47 (0.31–0.65) and standardized RMSE of 0.27 (0.19–0.38). Median (IQR) R^2^ and standardized RMSE for the worst models are 0.57 (0.44–0.67) and 0.32 (0.18–0.39) for conventional CV, and 0 (0–0.01) and 0.47 (0.31–0.62) for clustered CV.

### IEG model performance by pollutant

**Fig 3:**
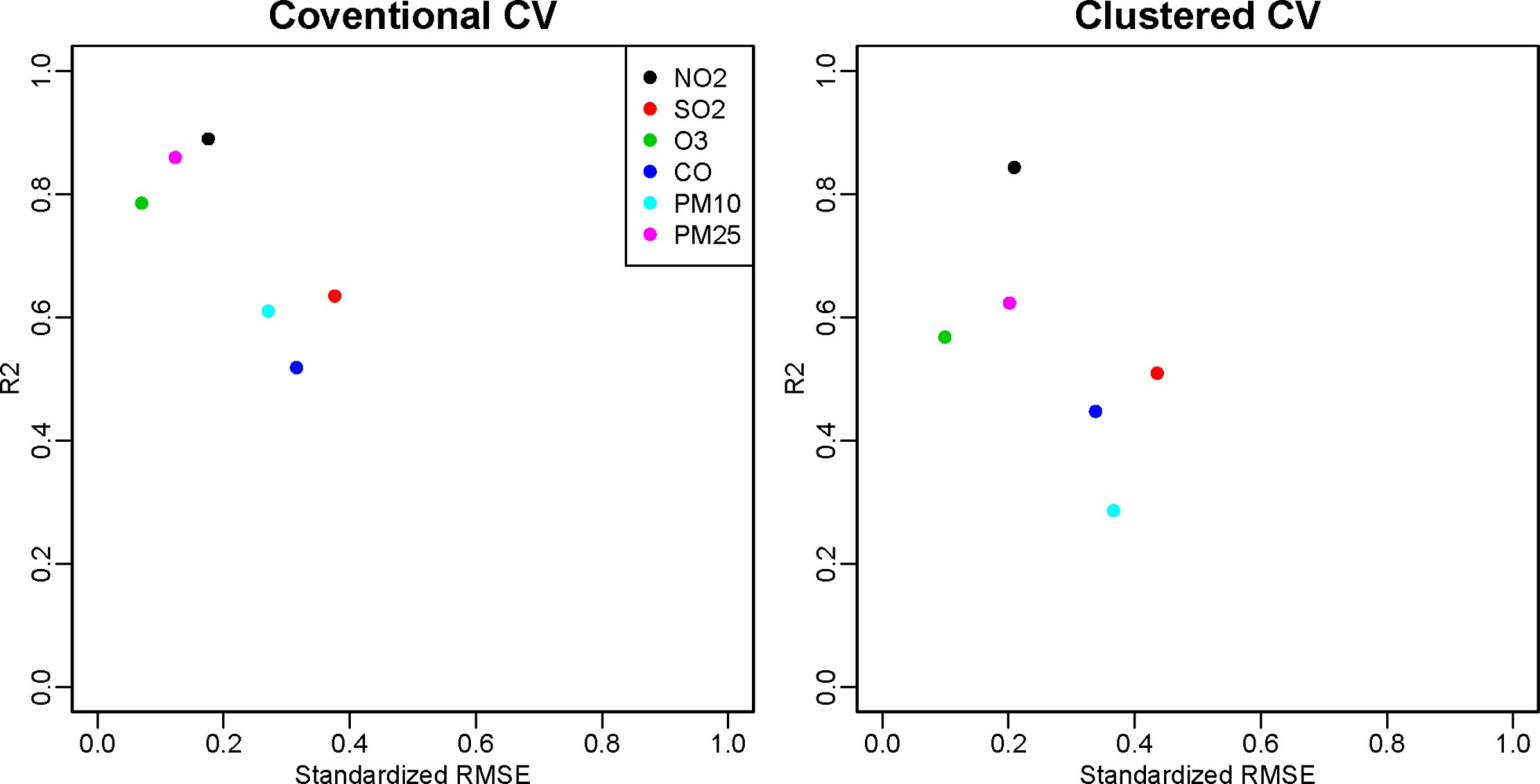
Standardized RMSEs and R^2^s from the “best” Integrated Empirical Geographic (IEG) models for the contiguous U.S. in 2000, for conventional and clustered cross-validation (CV), by pollutant.

Parsimonious models for PM_2.5_ and NO_2_ show generally good performance using conventional CV: median R^2^ (standardized RMSE) of the best models are 0.86 (0.13) for PM_2.5_ and 0.87 (0.21) for NO_2_ (Table 1, Fig 2). Analogous results using clustered CV are 0.65 (0.20) for PM_2.5_, and 0.80 (0.24) for NO_2_. For NO_2_, differences in model performance between “best” and “no variable” models are large particularly for clustered CV (median R^2^ for the best/no-variable model: 0.87/0.61 [conventional CV] and 0.80/0.00 [clustered CV]). That finding indicates the substantial benefit of having variables in the model when there are no monitors nearby and indicates that the ordinary kriging NO_2_ model including no variables offers nearly zero information far from monitors. In contrast, for SO_2_, ozone, and PM_10_, differences between “best” and “no variable” models are modest for conventional CV (median R^2^ for best/no-variable models: 0.59/0.57 [SO_2_], 0.75/0.72 [ozone], 0.59/0.49 [PM_10_]) (Fig 2 and S6 Fig). Analogous differences were larger for clustered CV (0.27/0.00 [SO_2_], 0.47/0.35 [ozone], 0.32/0.00 [PM_10_]). CO shows moderate model performance regardless of the number of variables (median R^2^ for best models: 0.47 [conventional CV] and 0.44 [clustered CV]) (S6 Fig). Overall, for both CV approaches, NO_2_ and PM_2.5_ yield better models than other pollutants (Fig 3). Over time, model performance tends to improve for ozone and PM_10_, decline for SO_2_ and CO, and remain relatively unchanged for PM_2.5_ and NO_2_.

### Selected variables

**Fig 4:**
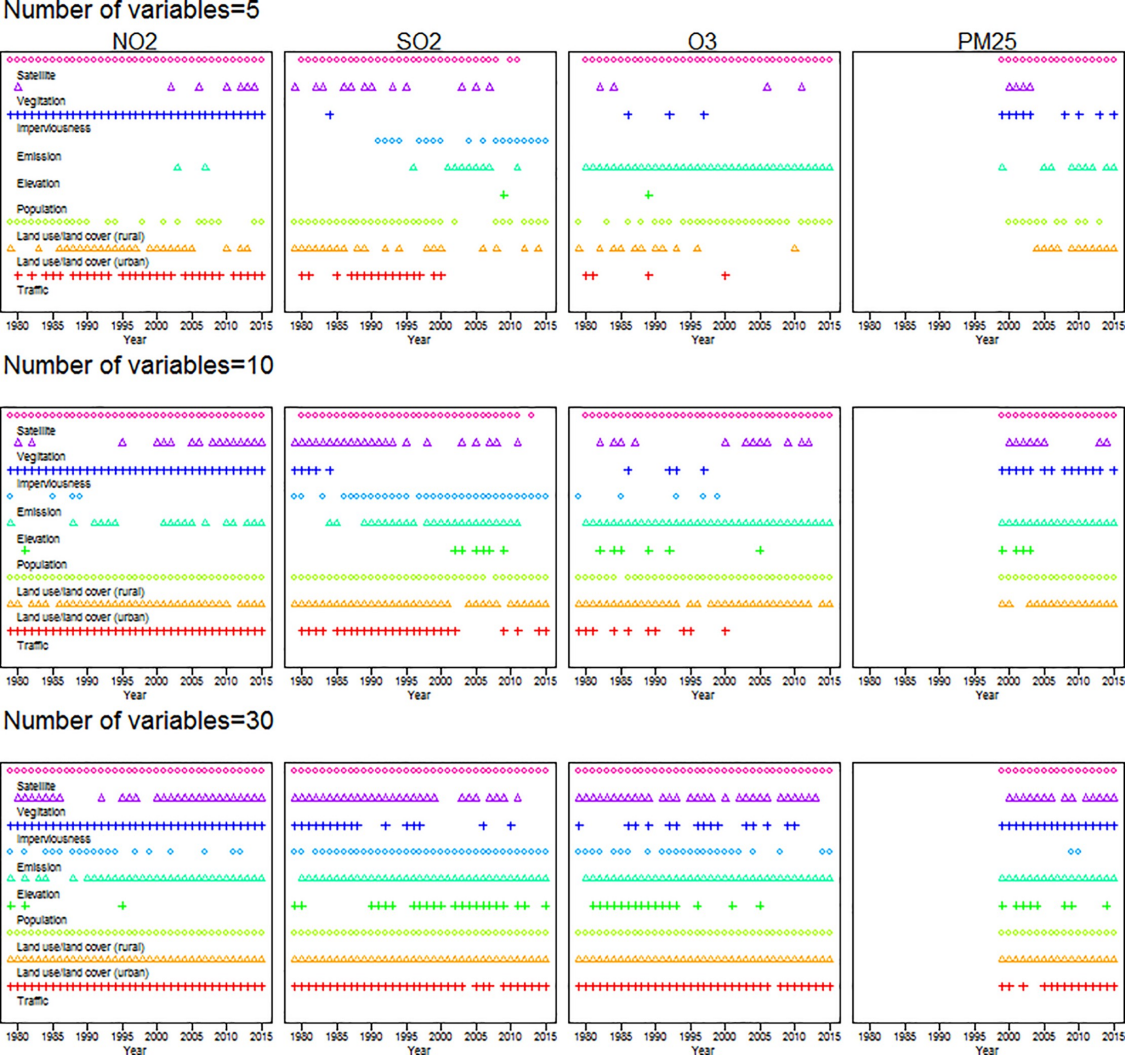
Categories (nine out of the eleven in S1 Table) of geographic variables chosen by forward selection for the national Integrated Empirical Geographic (IEG) models by year, pollutant (NO_2_, SO_2_, ozone, and PM_2.5_), and number of variables (5, 10, and 30) during 1979–2015 for the contiguous U.S.

Investigation of covariates by category chosen via forward selection (Fig 4 and S9 Fig) reveals that satellite air pollution estimates are almost always selected in the top 5 variables across all pollutants and years; urban or rural land use is consistently selected in the top 10 variables. Impervious surface and traffic are often selected for NO_2_, whereas emissions and/or elevation are common for SO_2_ and ozone, respectively. Models with the top 30 variables include almost all categories except population and emissions, depending on the year and pollutant.

### Sensitivity analyses

In our sensitivity analysis of re-computing CV statistics using conventional or clustered CV for the best and worst models selected based on the other CV approach, both CV approaches mostly give the identical best and worst models.

The three sensitivity analyses conducted on NO_2_ and PM_2.5_ for 2000 indicate the following. First, model performance is highly degraded when satellite variables are not included (S10 Fig), especially for clustered CV. The inclusion of land use variables becomes more important for models with larger numbers of variables. Second, when variable selection is random rather than via forward selection, model performance is noticeably reduced (S11 Fig). The performance gradually improves as more variables are added. However, even with random selection of variables, the improvement in performance for models with all variables relative to models with ~30 variables is small when using conventional CV. Thus, we find that even using a subset of randomly selected variables can yield models that are comparable to the “all variable” models. Third, when we shift the CV procedure to make it broader to include the entire model-building endeavor including forward selection rather than PLS and universal kriging, clustered CV results for NO_2_ show consistent patterns as with the core results of better performance with subsets of variables than all variables (S12 Fig). With clustered CV for PM_2.5_ and conventional CV for NO_2_ and PM_2.5_, expanding the aspects of modeling included in the CV procedure reduces the difference in model performance between “some variables” models and “all variables” models. CV statistics from conventional buffer-out CV give the similar pattern of better model performance using 3–30 variables but with mid-range CV statistics between conventional and clustered CVs (S13 Fig). In the comparison to the models using lasso for PLS, we found similar model performance with our approach using forward selection (S5 Table). Sensitivity analyses involving alternative metrics of ozone concentration reveal that our original approach using 8-hour moving averages during the summer season shows the best performance (S6 Table).

### Model application

**Fig 5:**
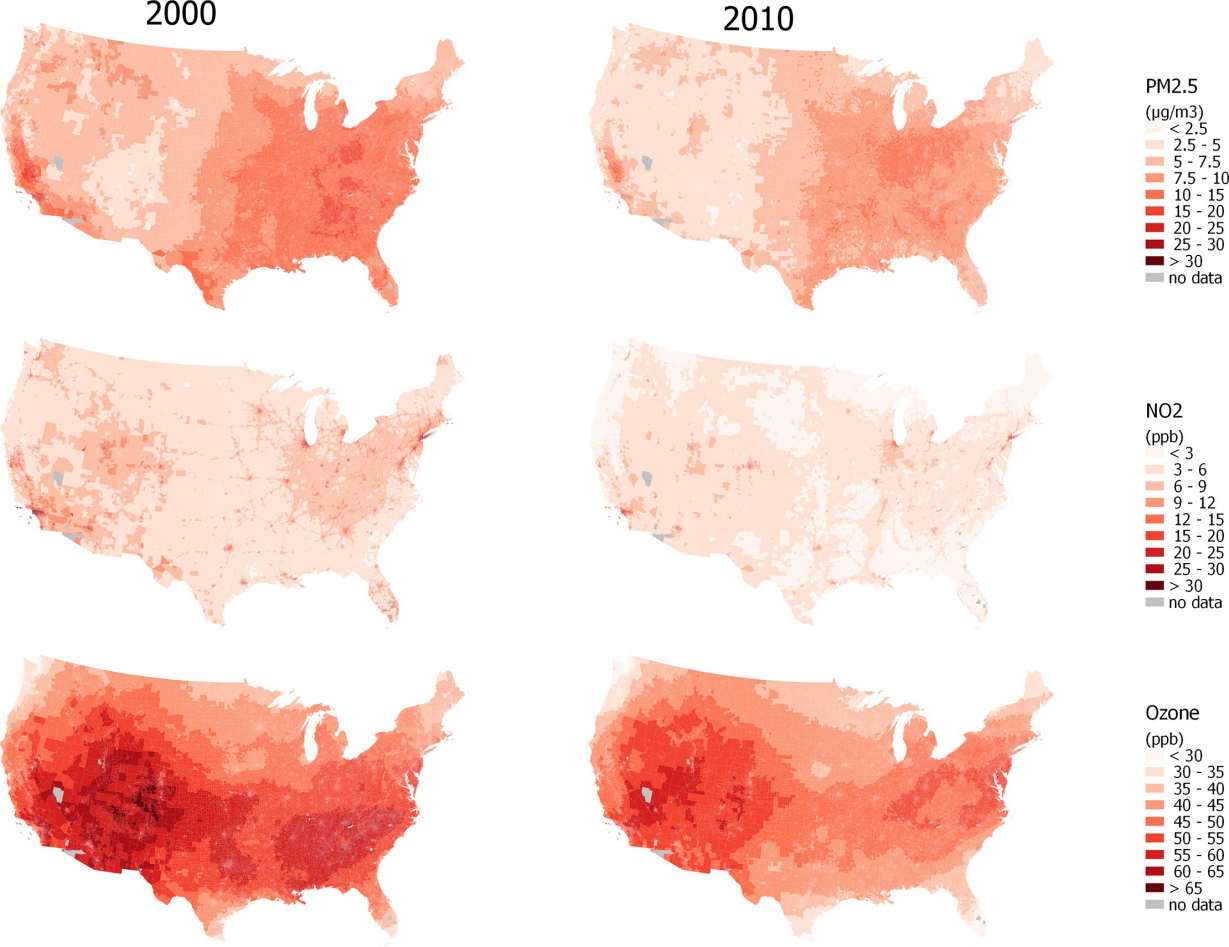
Maps of Census Block Group population-weighted mean predicted annual average concentrations for PM_2.5_, NO_2_, and ozone from the “best” national Integrated Empirical Geographic (IEG) models mostly including 3–30 variables for 2000 and 2010 in the contiguous U.S.

Predicted annual-average concentrations throughout the U.S. (Fig 5, and S14 and S15 Figs), generated using “best” models, reflect decreasing concentrations over 10–30 years depending on the pollutant. The extent of temporal change and the spatial patterns vary by pollutant. Population-weighted averages of annual average concentrations for Census Block Groups based on the predictions at Census Block centroids show similar means and narrow variability compared to those at monitoring sites (Table 2). Predicted concentrations and their uncertainties for all Block Groups, Tracts, Counties, and States in the contiguous U.S. are publicly and freely available online at https://www.caces.us.

**Table 2:** Summary statistics of population-weighted annual average concentrations across 215,491 Census Block Groups for the contiguous U.S., by pollutant and decadal year, based on predictions at Census Block centroids by using the “best” Integrated Empirical Geographic (IEG) models mostly using 3–30 geographic variables.

Pollutant	Year	Percentile					Mean	SD
		10	25	50	75	90		
NO_2_	1980	7.4	12.1	19.9	27.9	36.8	21.3	11.7
(ppb)	1990	6.1	8.4	12.9	19.0	26.9	15.2	9.2
	2000	5.6	7.8	11.8	16.7	23.2	13.3	7.5
	2010	3.3	4.7	7.2	10.8	15.8	8.5	5.1
SO_2_	1980	3.4	5.8	8.9	12.5	16.6	9.6	5.3
(ppb)	1990	2.0	3.0	4.6	7.0	9.2	5.3	3.0
	2000	1.8	2.2	3.1	4.4	6.1	3.6	1.8
	2010	0.9	1.2	1.5	2.0	2.5	1.6	0.7
Ozone	1980	39.0	45.4	51.3	57.3	63.6	51.1	9.6
(ppb)	1990	39.6	44.8	48.6	52.4	56.8	48.5	6.5
	2000	40.2	43.9	49.0	53.6	57.1	48.5	6.7
	2010	37.7	43.1	46.6	49.6	52.2	45.6	6.0
CO	1990	0.33	0.43	0.61	0.86	1.19	0.69	0.35
(ppm)	2000	0.29	0.35	0.43	0.55	0.74	0.48	0.20
	2010	0.23	0.28	0.31	0.35	0.39	0.31	0.07
PM_10_	1990	19.8	22.7	25.9	30.2	36.8	27.5	7.9
(μg/m^3^)	2000	15.7	18.8	22.0	25.4	30.8	22.9	6.8
	2010	12.8	15.2	18.3	21.5	24.1	18.4	4.6
PM_2.5_	2000	8.6	10.7	12.9	15.2	16.7	12.9	3.4
(μg/m^3^)	2010	6.3	7.9	9.6	10.8	12.1	9.4	2.2

## Discussion

We built and tested IEG models for six pollutants for all years with national monitoring data during 1979–2015 in the contiguous U.S.; predictions for “best-performing” models are publicly available online. We explore systematically the possibility of building parsimonious prediction models: how model performance changes when models are built by empirically selecting more or fewer variables from a large set to be incorporated in PLS dimension reduction.

Our findings indicate that models based on empirically selecting subsets of variables outperform or perform as well as “all variable” models: we find good model performance using a relatively small numbers of variables between 3 and 30 variables across pollutants and years. These findings suggest the good prediction ability of parsimonious prediction models when applied to prediction at unmeasured locations. In addition, satellite-derived estimates of air pollution and of land use were commonly-selected variables in the IEG models, indicating the importance of including these estimates to parsimonious models.

An important motivator for this research question is the considerable effort and computational intensity of tabulating hundreds of geographic variables at all prediction locations; that effort and computational intensity is a barrier to widespread usage of national IEG models. For example, in the U.S., the number of prediction locations could approach several thousand of people’s homes in a project-based cohort and more than million Block centroids in Census. This limitation impacts the feasibility of subsequent analyses in epidemiology, exposure assessment, environmental justice, and other fields. As the spatial domain for air pollution exposure models and health analyses is expanded to national or global scales [4,7,21,22,23,25], data and processing requirements will grow as additional input data are needed to improve prediction ability. Our approach reveals which predictive variables are most important for generating parsimonious prediction models that outperform or perform as well as all-variable models.

Model performance varied by pollutant, with better performance for PM_2.5_, NO_2_, and ozone than for CO, SO_2_, and PM_10_. All models benefited from introducing at least a small number of geographic covariates. Model performance is similar for ordinary kriging (with no variables) and IEG “best models” (mostly including 3–30 variables) for SO_2_ and to some extent for ozone when using conventional CV; using clustered CV, none of the pollutants exhibit similar performance between the “best” IEG and ordinary kriging models (although the gap is smallest for ozone). In general, ordinary kriging models deliver zero or near-zero value with clustered CV. This is sensible since kriging cannot predict to areas not incorporated in the model, as is required in the clustered CV.

Differences in model performance may reflect differences in chemistry and physics of the pollutants, spatial patterns of emissions, quality of input data, correlation with land uses, availability of relevant satellite data, and/or a design of monitoring networks (number of monitors and their placement). For example, the gap between ordinary kriging and “best” IEG is larger for NO_2_ than for PM_2.5_, reflecting spatial patterns are more homogeneous for PM_2.5_ than for NO_2_; and, the number of monitors is ~3× larger for PM_2.5_ than NO_2_. The extant monitoring network is designed for regulatory purposes: mainly, to test for compliance with National Ambient Air Quality Standards (NAAQS). As the use of IEG models grows, EPA or others could consider utility to IEG models (e.g., monitoring in locations with a variety of land uses) as an additional goal.

Some of our findings could have been sensitive to our methodological choices. However, results of our sensitivity analyses support our main findings. The slightly worse performance of the models using all variables as compared to models using some variables could potentially be driven by the fact that we treat the selected variables as fixed and do not include the selection process in our model evaluation. However, when we additionally include forward selection in our evaluation in one of our sensitivity analyses, the results are consistent with the original findings at least in clustered CV. Conventional CV shows similar performance across models using small versus all sets of variables. When we apply random selection instead of forward selection, more variables are needed; models including 50–150 variables, as opposed to 3–30 with forward selection, have performance similar to the all variable models. However, the minimal change in R^2^ suggests that some variable models still perform as adequately as the all variable models. In addition, good performance with reduced numbers of variables, consistently shown both in conventional and clustered CV, also supports good prediction ability of using subsets of variables, indicating the possibility of applying a parsimonious prediction approach to predict in areas without monitors. This finding also highlights the importance of clustered CV when evaluating observation-driven models. The relatively poor performance of clustered CV for some pollutants also suggests investigators should be careful in their application of prediction models to locally-affected pollutants when the monitoring network is spatially sparse.

Our results highlight the importance of satellite-derived air pollution data for IEG [20]; satellite data were selected as one or more of the top five variables consistently across all pollutants and years. The most commonly selected satellite estimates were satellite PM_2.5_ derived from AOD data for PM_2.5_ models and HCHO for ozone models. Considering IEG model performance when a category of variables is excluded, the performance decline was greatest excluding satellite data in comparison to excluding other covariate data, especially with clustered CV.

A common concern for IEG models such as those generated here is whether regulatory monitoring data can allow our model to provide accurate predictions for people. The distribution of values for geographic variables such as land use characteristics might differ at monitoring locations relative to prediction locations where people live [41,42]. Monitors may be located in areas where few people live and may not be able to represent people’s exposures. When we compared the distribution of geographic variables between all available regulatory monitoring sites and Census Block centroids, for 95% and 98% of ~350 variables, the standard deviation for Census Block centroids is 2.5 and 5 times standard deviation for monitoring sites. This finding indicates that the range of values at Block centroids exceeds the range at monitoring sites, suggesting that there could be some spatial misalignment between monitoring and prediction locations in our work. However, because our models use estimated PLS predictors instead of direct measures of variables, extreme values of a few variables are less likely to impact model predictions [37].

Although we focus on investigating the impact of model parsimony on model performance within the IEG framework instead of between IEG and other modeling approaches, our IEG models provide consistent model performance when compared to previous studies. The IEG models using some to all geographic variables for 2000–2010 shows cross-validated R^2^s of 0.84–0.86 for PM_2.5_ and 0.81–0.87 for NO_2_, which are similar to estimates from other studies in the contiguous U.S. for the overlapping periods: 0.78–0.88 for PM_2.5_ in neural network and 0.78–0.82 for NO_2_ in land use regression [4,21,23]. We did not use direct measures of variables. In a previous study [37], using selected variables alone gave extreme predictions at a few subject locations although the model performance based on monitoring data was similar. These extreme predictions occurred because of widely different geographic characteristics at subject homes from those at monitoring sites. This earlier finding suggested a caution when we use a few selected geographic variables instead of summary predictors estimated by dimension reduction methods. In the current study (and as would be expected in most studies), the distribution of values for geographic variables at the census tract centroids is generally wider than that of monitoring sites, indicating the possibility of producing inaccurate health effect estimates when direct measures of variables are used. As another alternative approach, our future work will compare this IEG model and a machine learning approach based on the same input data.

The incorporation of variable selection into PLS regression was widely applied in previous studies [38] but rarely recognized in environmental epidemiology. For example, genetic studies used these approaches to identify the list of genetic characteristics related to health outcomes from numerous genetic information. These combined approaches of variable selection to PLS select the variables based on PLS properties such as PLS loadings and scores, or iterative procedures between variable selection and PLS model fitting. Although there is a similarity with respect to combining variable selection and PLS between those approaches and ours, there are two major distinctions. First, the combined approaches test subsets of variables in PLS based on good understanding of model parsimony. In genetic analyses where most genetic variables are irrelevant to outcome such as disease status, there is a strong consensus that adding noisy variables impairs model performance. In contrast, hundreds of geographic variables computed for air pollution prediction are considered as potential pollution sources and being related to air pollution, and previous studies have expanded lists of variables. Secondly, based on little knowledge of benefit of including a large set of geographic variables, our aim of using the combined approach was the investigation of model parsimony for prediction rather than the selection of a small subset of variables. Our finding of better model performance using a small subset of variables was not previously investigated in air pollution studies.

Our study has several limitations to motivate future research. We consider only spatial aspects of IEG models and use many temporally-fixed geographic variables (exceptions include satellite-derived estimates of air pollution concentrations, and land use variables for the 1970s and 2006). Future studies could also add variables that represent geographic characteristics changing over time. In addition, temporal correlation over years also can contribute to prediction when we develop a prediction model in a spatio-temporal framework. Future work could build national, publicly available models with finer temporal resolution than here (i.e., better than annual-averages) and could test model parsimony with respect to temporal models or spatiotemporal models. As alternative modeling approaches, non-linear PLS and additive models could be considered. Other modeling approaches particularly for various machine learning approaches applied in recent studies should be investigated [4,29]. Satellite air pollution estimates employed here for NO_2_, SO_2_, and HCHO are tropospheric column abundance, rather than ground-level estimates. Previous studies have shown that NO_2_ model improvements from satellite-derived estimates of air pollution are similar for column-total as for ground-level estimates [21,43]; future work could test that finding for SO_2_ and HCHO. The present research employed emission estimates, which are an input into chemical transport models (CTMs), and prior research has included CTM as an input to IEG model-building [4,44]. Future research could test the role of model parsimony in IEGs that incorporate CTM output. Future IEG models could also potentially include national datasets on traffic volumes, vehicle fleet composition, enhanced urban form estimates from Landsat imagery or point-of-interest data, and recently-launched satellites. We hypothesize that such datasets would improve IEG model performance, though recognizing that because the IEG models already have many inputs (including satellite-based estimates of air pollution concentrations), new datasets may or may not improve model performance appreciably. For the variables characterized as summaries within circular buffers of several distances, transport network distance can be an alternative approach for the current Euclidian distance. Lastly, we found that the performance of the models with the randomly selected subsets of variables, although we need more variables than in forward selection, was as good as the models using the full set. This may indicate high correlation between many variables. Future studies need further investigation to confirm this finding.

In summary, this study provides important findings on cost-effective approaches for national-scale air pollution prediction. Results indicate that national IEG model performance can be similar or better, depending on the pollutant and evaluation approach, if built on only a small number of empirically selected covariates from hundreds, relative to models built using all of those variables. This finding suggests good applicability of parsimonious models to predicting at any locations in a country. Our model predictions for the contiguous U.S. are freely available online, at https://www.caces.us.

## Supporting information

S1 File (DOCX)

S1 Table List of geographical variables and satellite air pollution estimates. (DOCX)

S2 Table Available years of satellite estimates for air pollution and metrics used for national prediction models. (DOCX)

S3 Table Summary statistics of annual average concentrations for six criteria air pollutants across regulatory monitoring sites in the contiguous U.S. for 1980, 1990, 2000, and 2010. (DOCX)

S4 Table Medians of estimated covariance parameters and proportions of predictions explained by PLS regression across the national Integrated Empirical Geographic (IEG) models including different numbers of variables for NO_2_ and PM_2.5_ in 2000. (DOCX)

S5 Table CV statistics of IEG models for annual average concentrations of NO_2_ and PM_2.5_ in 2000 using PLS predictors estimated from subsets of geographic variables and using the selected variables without PLS. (DOCX)

S6 Table Cross-validation (CV) statistics of the ozone national Integrated Empirical Geographic (IEG) models including no some and all geographic variables and/or satellite estimates for four metrics by summer and all seasons. (DOCX)

S1 Fig Numbers of regulatory monitoring sites that meet our site inclusion criteria for computing representative annual average concentrations of six criteria air pollutants for 1979–2015 in the continental U.S. (DOCX)

S2 Fig Diagram of data preprocessing and variable computation for more than 900 geographic variables. (DOCX)

S3 Fig Numbers of geographic variables retained after excluding least informative variables by pollutant and year. (DOCX)

S4 Fig Map of 10 spatial clusters of 345 NO_2_ regulatory monitoring sites in 2000 determined by k-means clustering. (DOCX)

S5 Fig The relationship between numbers of variables and cross-validation (CV) statistics from the national Integrated Empirical Geographic (IEG) models of six criteria air pollutants in 2000 by conventional and clustered cross-validation (vertical lines for 10, 30, and 60). (DOCX)

S6 Fig Standardized RMSEs and R2s of the national Integrated Empirical Geographic (IEG) models including no, some, and all variables from conventional and clustered cross-validation (CV) during 1979–2015 for the contiguous U.S. by CO and PM_10_. (DOCX)

S7 Fig Numbers of variables selected for the best and worst national Integrated Empirical Geographic (IEG) models with the highest and lowest cross-validated R^2^s, respectively (which was also the model with the lowest and highest standardized root mean square error), by pollutant and CV type (conventional CV, clustered CV). For ease of reading, figures include horizontal lines for y-axis values of 30, 50, and 100.
(DOCX)

S8 Fig Standardized root mean square errors and R^2^s of the national Integrated Empirical Geographic (IEG) models including no variables, some variables (i.e., between 3 and 30 variables), and all variables from conventional and clustered cross-validation, by year and pollutant, for the contiguous U.S. (DOCX)

S9 Fig Categories (nine out of the eleven in S1 Table) of geographic variables chosen by forward selection for the national Integrated Empirical Geographic (IEG) models by year, pollutant (CO and PM_10_), and number of variables (5, 10, and 30) during 1979–2015 for the contiguous U.S. (DOCX)

S10 Fig The relationship between numbers of variables and cross-validation (CV) statistics from the national Integrated Empirical Geographic (IEG) models of NO_2_ and PM_2.5_ in 2000 by exclusion of a different category of geographic variables and satellite air pollution estimates by conventional and clustered cross-validation. For ease of reading, vertical lines are shown at x-axis values of 10, 30, and 60.
(DOCX)

S11 Fig The relationship between the same numbers of randomly selected variables as those in forward selection and cross-validation (CV) statistics from the national Integrated Empirical Geographic (IEG) models of NO_2_ and PM_2.5_ in 2000 by conventional and clustered cross-validation. For easy of viewing, vertical lines are shown at x-axis values of 10, 30, and 60.
(DOCX)

S12 Fig The relationship between numbers of variables and cross-validation (CV) statistics to compare including forward selection and estimation of PLS predictors, with parameter estimation alone in the national Integrated Empirical Geographic (IEG) models of NO_2_ and PM_2.5_ in 2000 by conventional and clustered CV. Vertical lines shown for x-axis values of 10, 30, and 60.
(DOCX)

S13 Fig The relationship between numbers of variables and cross-validation (CV) statistics from conventional, clustered, and conventional buffer-out CVs in the national Integrated Empirical Geographic (IEG) models of NO_2_ and PM_2.5_ in 2000. (DOCX)

S14 Fig Maps of Census Block Group population-weighted mean for PM_10_, CO, and SO_2_ from the “best” Integrated Empirical Geographic (IEG) models mostly including 3–30 geographic variables for 2000 and 2010 in the contiguous U.S. (DOCX)

S15 Fig Quantile-based plots of population-weighted annual average concentrations of six criteria air pollutants across 215,491 Census Block Group centroids, based on predicted concentrations at Census Block centroids by using the “best” Integrated Empirical Geographic (IEG) models mostly using 3–30 geographic variables for 1979–2015 in the contiguous U.S. (DOCX)

## Abbreviation

AOD: Aerosol Optical Depth
CO: Carbon Monoxide
CTM: Chemical Transport Model
CV: Cross-validation
EPA: Environmental Protection Agency
HCHO: Formaldehyde
IEG: Integrated Environmental Geographic
LUR: Land Use Regression
NO2: Nitrogen Dioxide
PLS: Partial Least Squares
PM2.5: Particulate Matter less than or equal to 2.5 micron per diameter
PM10: Particulate Matter less than or equal to 10 micron per diameter
R2: R-squared
RMSE: Root Mean Square Error
SO2: Sulfur dioxide
UK: Universal Kriging

## References

[1] Eeftens M, Beelen R, de Hoogh K, Bellander T, Cesaroni G, Cirach M, et al Development of land use regression models for PM(2.5), PM(2.5) absorbance, PM(10) and PM(coarse) in 20 European study areas; results of the ESCAPE project. Environ. Sci. Technol. 2012;46(20):11195–11205. 10.1021/es301948k 22963366

[2] Keller JP, Olives C, Kim SY, Sheppard L, Sampson PD, Szpiro AA, et al A unified spatiotemporal modeling approach for prediction of multiple air pollutants in the multi-ethnic study of atherosclerosis and air pollution. Environ. Health Perspect. 2015;123(4):301–309. 10.1289/ehp.1408145 25398188 PMC4384200

[3] Kim SY, Olives C, Sheppard L, Sampson PD, Larson TV, Keller JP, et al Historical prediction modeling approach for estimating long-term concentrations of PM_2.5_ in cohort studies before the 1999 implementation of widespread monitoring. Environ Health Perspect. 2017;125(1):38–46. 10.1289/EHP131 27340825 PMC5226688

[4] Di Q, Kloog I, Koutrakis P, Lyapustin A, Wang Y, Schwartz J. Assessing PM_2.5_ exposures with high spatiotemporal resolution across the continental United States. Environ Sci Technol. 2016;50:4712–4721. 10.1021/acs.est.5b06121 27023334 PMC5761665

[5] Knibbs LD, van Donkelaar A, Martin RV, Bechle MJ, Brauer M, Cohen D, et al Satellite-based land-use regression for continental-scale long-term ambient PM2.5 exposure assessment in Australia. Environ Sci Technol. 2018;52(21):12445–12455. 10.1021/acs.est.8b02328 30277062

[6] Ma Z, Hu X, Huang L, Bi J, Liu Y. Estimating Ground-Level PM_2.5_ in China Using Satellite Remote Sensing. Environ Sci Technol. 2014;48(13):7436–7444. 10.1021/es5009399 24901806

[7] van Donkelaar A, Martin RV, Brauer M, Hsu NC, Kahn RA, Levy RC, et al Global estimates of fine particulate matter using a combined geophysical-statistical method with information from satellites, models, and monitors. Environ Sci Technol. 2016;50(7):3762–3772. 10.1021/acs.est.5b05833 26953851

[8] Beelen R, Raaschou-Nielsen O, Stafoggia M, Andersen ZJ, Weinmayr G, Hoffmann B, et al Effects of long-term exposure to air pollution on natural-cause mortality: an analysis of 22 European cohorts within the multicenter ESCAPE project. Lancet. 2014;383(9919):785–795. 10.1016/S0140-6736(13)62158-3 24332274

[9] Di Q, Wang Y, Zanobetti A, Wang Y, Koutrakis P, Choirat C, et al Air pollution and mortality in the Medicare population. N Engl J Med. 2017a;376(26):2513–2522. 10.1056/NEJMoa1702747 28657878 PMC5766848

[10] Kaufman JD, Adar SD, Barr RG, Budoff M, Burke GL, Curl CL, et al Association between air pollution and coronary artery calcification within six metropolitan areas in the USA (the Multi-Ethnic Study of Atherosclerosis and Air Pollution): a longitudinal cohort study. Lancet. 2016;13(10045):696–704. 10.1016/S0140-6736(16)00378-0 27233746 PMC5019949

[11] Yin P, Brauer M, Cohen A, Burnett RT, Liu J, Liu Y, et al Long-term fine particulate matter exposure and nonaccidental and cause-specific mortality in a large national cohort of Chinese men. Environ Health Perspect. 2017;125(11):117002 10.1289/EHP1673 29116930 PMC5947939

[12] Fann N, Kim SY, Olives C, Sheppard L. Estimated changes in life expectancy and adult mortality resulting from declining PM_2.5_ exposures in the contiguous United States: 1980–2010. Environ Health Perspect. 2017;125(9):097003 10.1289/EHP507 28934094 PMC5903877

[13] Xie Y, Dai H, Dong J, Hanaoka T, Masui T. Economic Impacts from PM2.5 Pollution-Related Health Effects in China: A Provincial-Level Analysis. Environ Sci Technol. 2016;50(9):4836–43. 10.1021/acs.est.5b05576 27063584

[14] Clark LP, Millet DB, Marshall JD. Changes in Transportation-Related Air Pollution Exposures by Race-Ethnicity and Socioeconomic Status: Outdoor Nitrogen Dioxide in the United States in 2000 and 2010. Environ Health Perspect. 2017;125(9):097012 10.1289/EHP959 28930515 PMC5915204

[15] Hajat A, Diez-Roux AV, Adar SD, Auchincloss AH, Lovasi GS, O’Neill MS, et al Air pollution and individual and neighborhood socioeconomic status: evidence from the multi-ethnic study of atherosclerosis (MESA). Environ Health Perspect. 2013;121(11–12):1325–1333. 10.1289/ehp.1206337 24076625 PMC3855503

[16] Hoek G, Beelen R, de Hoogh K, Vienneau D, Gulliver J, Fischer P, et al A review of land-use regression models to assess spatial variation of outdoor air pollution. Atmos Environ. 2008;2(33): 7561–7578. 10.1016/j.atmosenv.2008.05.057

[17] de Hoogh K, Gulliver J, Donkelaar AV, Martin RV, Marshall JD, Bechle MJ, et al Development of West-European PM_2.5_ and NO_2_ land use regression models incorporating satellite-derived and chemical transport modelling data. Environ Res. 2016;151:1–10. 10.1016/j.envres.2016.07.005 27447442

[18] Wang M, Sampson PD, Hu J, Kleeman M, Keller JP, Olives C, et al Combining Land-Use Regression and Chemical Transport Modeling in a Spatiotemporal Geostatistical Model for Ozone and PM_2.5_. Environ Sci Technol. 2016;17;50(10):5111–5118. 10.1021/acs.est.5b06001 27074524 PMC5096654

[19] Chu Y, Liu Y, Li X, Liu Z, Lu H, Lu Y, et al A review on predicting ground PM2.5 concentration using satellite aerosol optical depth. Atmosphere. 2016;7(129):1–25. 10.3390/atmos710012927088005

[20] Hoek G. Methods for Assessing Long-Term Exposures to Outdoor Air Pollutants. Curr Environ Health Rep. 2017;4(4):450–462. 10.1007/s40572-017-0169-5 29064065 PMC5676801

[21] Bechle M, Millet DB, Marshall JD. National spatiotemporal exposure surface for NO_2_: monthly scaling of a satellite-derived land-use regression, 2000–2010. Environ Sci Technol. 2015;49(20):12297–12305. 10.1021/acs.est.5b02882 26397123

[22] Larkin A, Geddes JA, Martin RV, Xiao Q, Liu Y, Marshall JD, et al Global land use regression model for nitrogen dioxide air pollution. Environ Sci Technol. 2017;51 (12):6957–6964. 10.1021/acs.est.7b01148 28520422 PMC5565206

[23] Novotny EV, Bechle M, Millet DB, Marshall JD. National satellite-based land-use regression: NO_2_ in the United States. Environ Sci.Technol. 2011;45(10):4407–4414. 10.1021/es103578x 21520942

[24] Xu H, Bechle MJ, Wang M, Szpiro AA, Vedal S, Bai Y, et al National PM_2.5_ and NO_2_ exposure models for China based on land use regression, satellite measurements, and universal kriging. Sci Total Environ. 2018;12;655:423–433. 10.1016/j.scitotenv.2018.11.125 30472644

[25] Young MT, Bechle MJ, Sampson PD, Szpiro AA, Marshall JD, Sheppard L, et al Satellite-Based NO_2_ and Model Validation in a National Prediction Model Based on Universal Kriging and Land-Use Regression. Environ Sci Technol. 2016;50(7):3686–94. 10.1021/acs.est.5b05099 26927327 PMC5104568

[26] Mercer LD, Szpiro AA, Sheppard L, Lindström J, Adar SD, Allen RW, et al Comparing universal kriging and land-use regression for predicting concentrations of gaseous oxides of nitrogen (NOx) for the Multi-Ethnic Study of Atherosclerosis and Air Pollution (MESA Air). Atmos Environ. 2011;45(26):4412–4420. 10.1016/j.atmosenv.2011.05.043 21808599 PMC3146303

[27] Sampson PD, Richards M, Szpiro AA, Bergen S, Sheppard L, Larson TV, et al A regionalized national universal kriging model using partial least squares regression for estimating annual PM2.5 concentrations in epidemiology. Atmos Environ. 2013;75:383–392. 10.1016/j.atmosenv.2013.04.015 24015108 PMC3763950

[28] Beckerman BS, Jerrett M, Serre M, Martin R, Lee SJ, van Donkelaar A, et al A hybrid approach to estimating national scale spatiotemporal variability of PM_2.5_ in the contiguous United States. Environ Sci Technol. 2013;47(13):7233–7241. 10.1021/es400039u 23701364 PMC3976544

[29] Di Q, Rowland S Koutrakis P, Schwartz J. A hybrid model for spatially and temporally resolved ozone exposures in the continental United States. J Air Waste Manag Assoc. 2017b;67(1): 39–52. 10.1080/10962247.2016.1200159 27332675 PMC5741295

[30] Boersma KF, Eskes HJ, Dirksen RJ, van der A RJ, Veefkind JP, Stammes P, et al 2011 An improved retrieval of tropospheric NO2 columns from the Ozone Monitoring Instrument. Atmos Meas Tech 4:1905–1928. 10.5194/amt-4-1905-2011

[31] Kelly C. 2007 OMI/Aura Formaldehyde (HCHO) Total Column 1-orbit L2 Swath 13x24 km V003, Greenbelt, MD, USA, Goddard Earth Sciences Data and Information Services Center (GES DISC), 10.5067/Aura/OMI/DATA2015 (https://gcmd.nasa.gov/KeywordSearch/Metadata.do?Portal=GCMD&KeywordPath=%5BSource_Name%3A+Short_Name%3D%27AURA%27%5D&OrigMetadataNode=GCMD&EntryId=OMHCHO_003&MetadataView=gwt&MetadataType=0&lbnode=mdlb1).

[32] OMI Science Team. 2012 OMI/Aura Level 2 Sulphur Dioxide (SO2) Trace Gas Column Data 1-Orbit subset Swath along CloudSat track 1-Orbit Swath 13x24 km, Edited by GES DISC, Greenbelt, MD, USA, Goddard Earth Sciences Data and Information Services Center (GES DISC) (https://disc.gsfc.nasa.gov/datacollection/OMSO2_CPR_003.html).

[33] Deeter MN, Edwards DP, Francis GL, Gille JC, Martinez-Alonso S, Worden HM, et al 2017 A Climate-scale satellite record for carbon monoxide: the MOPITT version 7 product. Atmos Meas Tech 10:2533–2555. 10.5194/amt-10-2533-2017

[34] De Smedt I, Müller JF, Stavrakou T, van der A R, Eskes H, Van Roozendael M. 2018 Twelve years of global observations of formaldehyde in the troposphere using GOME and SCIAMACHY sensors. Atmos Chem Phys 8(16):4947−4963. 10.5194/acp-8-4947-2008

[35] Bergen S, Sheppard L, Sampson PD, Kim SY, Richards M, Vedal S, et al A national prediction model for components of PM_2.5_ and measurement error corrected health effect inference. Environ. Health Perspect. 2013;121(9):1017–1025. 10.1289/ehp.1206010 23757600 PMC3764074

[36] Banerjee S, Carlin BP, Gelfand AE. Basics of point-referenced data models In: Hierarchical Modeling and Analysis for Spatial Data. Boca Raton, FL: Chapman & Hall/CRC Press; 2004 pp. 21–68.

[37] Mehmood T, Liland KH, Snipen L, Sæbø S. A review of variable selection methods in Partial Least Squares Regression. Chemometrics and Intelligent Laboratory Systems. 2012;118:62–69. 10.1016/j.chemolab.2012.07.010.

[38] Kim SY, Sheppard L, Bergen S, Szpiro AA, Sampson PD, Kaufman JD, et al Prediction of fine particulate matter chemical components with a spatio-temporal model for the Multi-Ethnic Study of Atherosclerosis cohort. J Exp Sci Environ Epidemiol. 2016;26(5):520–528. 10.1038/jes.2016.29 27189258 PMC5104659

[39] Roberts DR, Bahn V, Ciuti S, Boyce MS, Elith J, Guillera-Arroita G, et al Cross-validation strategies for data with temporal, spatial, hierarchical, or phylogenetic structure. Ecography. 2017;40(8):913–929. 10.1111/ecog.02881.

[40] Tibshirani R. The lasso Method for Variable Selection in the Cox Model". Statistics in Medicine. 1997;16(4):385–395. 10.1002/(sici)1097-0258(19970228)16:4<385::aid-sim380>3.0.co;2-3 9044528

[41] Szpiro AA, Paciorek CJ, Sheppard L. Does more accurate exposure prediction necessarily improve health effect estimates? Epidemiology. 2011;22(5);680–685. 10.1097/EDE.0b013e3182254cc6 21716114 PMC3195520

[42] Szpiro AA and Paciorek CJ. Measurement error in two-stage analyses, with application to air pollution epidemiology. Environmetrics. 2013;24;501–517. 10.1002/env.2233 24764691 PMC3994141

[43] Knibbs LD, Hewson MG, Bechle MJ, Marshall JD, Barnett AG. A national satellite-based land-use regression model for air pollution exposure assessment in Australia. Environ Res. 2014 135:204–211. 10.1016/j.envres.2014.09.011 25282278

[44] Wang M, Gehring U, Hoek G, Keuken M, Jonkers S, Beelen R, et al Air Pollution and Lung Function in Dutch Children: A Comparison of Exposure Estimates and Associations Based on Land Use Regression and Dispersion Exposure Modeling Approaches. Environ. Health Perspect. 2015;123(8):847–851. 10.1289/ehp.1408541 25839747 PMC4529005

