# Supplementary material for: Concentrations of criteria pollutants in the contiguous U.S., 1979 – 2015: Role of prediction model parsimony in integrated empirical geographic regression
Source: PLoS One. 2020 Feb 18;15(2):e0228535. doi: 10.1371/journal.pone.0228535 (PMC7028280; doi:10.1371/journal.pone.0228535)
Supplement: S1 File — (DOCX) [file pone.0228535.s001.docx]

**Supporting information**

Concentrations of criteria pollutants in the contiguous U.S., 1979 – 2015: Role of model parsimony in integrated empirical geographic regression

Sun-Young Kim, Matthew Bechle, Steve Hankey, Lianne Sheppard, Adam A. Szpiro, Julian D. Marshall.

Contents

Data generation and processing for satellite air pollution estimates

Model equation

R code to predict annual-average air pollution concentrations using the Integrated Empirical Geographic (IEG) model

Tables S1-S6

Figure S1-S15

**Data generation and processing for satellite air pollution estimates**

PM_2.5_: We employ previously published, publicly available, satellite-based estimates of annual-average ground-level PM_2.5_ for years 1998-2014 on a 0.1° grid. Briefly, five aerosol optical depth (AOD) satellite retrievals (from several instruments and retrieval algorithms) are combined with (1) satellite-based measurements of vertical aerosol profiles, (2) modeled AOD and ground-level PM_2.5_ from a global chemical transport model (GEOS-Chem), and (3) ground-based AOD measurements from the aerosol robotic network (AERONET) to estimate annual ground-level PM_2.5_ on a 0.1° grid (van Donkelaar et al., 2016).

CO: We obtain daily L2 (i.e., processed data at native instrument resolution) surface-level CO multispectral (combined near infrared and thermal infrared) retrievals (v7) from the Measurements of Pollution in The Troposphere (MOPITT) sensor on the National Aeronautics and Space Administration (NASA)’s Terra satellite for years 2001-2016 (Deeter et al., 2017). For each year, daily surface-level CO measurements are screened for missing data and solar zenith angle (SZA) >80°, then oversampled onto a 0.25° × 0.25° grid. Oversampling is an averaging method for satellite data that takes advantage of overlapping pixels when temporally averaging measurements at native resolution; all pixels falling within a circular buffer centered on each grid cell are averaged to that grid cell.

NO_2_: We obtain daily L2 (native instrument resolution) tropospheric NO_2_ retrievals from the Ozone Monitoring Instrument (OMI) onboard the NASA Earth Observing System (EOS)-Aura satellite (DOMINOv2) for years 2005-2015 from the Tropospheric Emission Monitoring Internet Service ([www.temis.nl](http://www.temis.nl)) (Boersma et al., 2011). The L2 NO_2_ data are screened for missing data, flags (including “row anomaly” flags: see http://projects.knmi.nl/omi/research/product/rowanomaly-background.php), SZA > 60°, cloud fraction >40%, and surface albedo >30%. For each year and for 3-year averages, screened daily tropospheric NO_2_ are oversampled onto a 0.1° × 0.1° grid.

HCHO: We obtain daily L2 tropospheric HCHO retrievals from OMI onboard the NASA EOS-Aura satellite for years 2005-2016 from NASA’s Goddard Earth Sciences Data and Information Services Center (GES-DISC) (Kelly 2007). Daily L2 HCHO data are screened using the same criteria as the L2 NO2 retrievals above. HCHO is more difficult to detect from space than NO2, owing to a lower signal-to-noise ratio and spectral interference from other molecules in the same fitting window (De Smedt et al., 2008); we therefore oversample screened daily tropospheric HCHO for the entire 12 year period (2005-2016) onto a 0.25° × 0.25° grid.

SO_2_: We obtain daily L3 (pre-gridded product) tropospheric SO_2_ retrievals from OMI onboard the NASA EOS-Aura satellite for years 2005-2016 from NASA’s GES-DISC (OMI Science Team 2012). Daily gridded 0.25° × 0.25° L3 SO_2_ data are screened for data flags (including “row anomaly”) and temporally averaged to annual averages.

Using the gridded products described above, we assign to each target location annual or long-term averages of daily observations (Table S2). For the years before or after the satellite data are available, we use the average of the 3 closest years.

**Model equation**

Using the universal kriging model, we hypothesize that the annual average concentration of an air pollutant at a location s ($C\left( s \right)$) is comprised of the mean component ($\beta_{0}+\sum_{j=1}^{m} \beta_{j}X_{j}(s)$) and the variance component ($\varepsilon\left( s \right)$), as shown in the equation below.

$$C\left( s \right)=\beta_{0}+\sum_{j=1}^{m} \beta_{j}X_{j}(s)+\varepsilon\left( s \right)$$

$$\varepsilon\left( s \right)\sim N(0, \Sigma\left( \phi,\sigma^{2},\tau^{2} \right))$$

The mean component includes two or three (j= 1 to m; m=2 or 3) summary predictors ($X_{j}(s)$) estimated by partial least squares. The variance component is parameterized by three covariance parameters: the range ($\phi$), partial sill ($\sigma^{2}$), and nugget ($\tau^{2}$) which represent the spatial correlation distance, spatial variability, and non-spatial variability, respectively. The regression and covariance parameters were estimated by the maximum likelihood method. Further details of our approach builds on a universal kriging framework were described elsewhere (Bergen et al. 2013; Sampson et al. 2013; Young et al. 2013).

[References]

Bergen S, Sheppard L, Sampson PD, Kim SY, Richards M, Vedal S, et al. A national prediction model for components of PM2.5 and measurement error corrected health effect inference. Environ. Health Perspect. 2013;121(9).

Sampson PD, Richards M, Szpiro AA, Bergen S, Sheppard L, Larson TV, et al. A regionalized national universal kriging model using partial least squares regression for estimating annual PM2.5 concentrations in epidemiology. Atmos Environ. 2013;75:383–392.

Young MT, Bechle MJ, Sampson PD, Szpiro AA, Marshall JD, Sheppard L, et al. Satellite-Based NO2 and Model Validation in a National Prediction Model Based on Universal Kriging and Land-Use Regression. Environ Sci Technol. 2016;50(7):3686-94. .

**R code to predict annual-average air pollution concentrations using the Integrated Empirical Geographic (IEG) model**

library(pls)

library(geoR)

library(data.table)

nat.pred <- function( model.obj, n.var, covar.pred, nchunk=500 ){

desc.vars <- c('county','state','state_plane', 'lambert_x','lambert_y')

# 1. monitoirng data and modeling objects

covars <- model.obj$covars

pls <- model.obj$pls

geodata <- model.obj$geodata

fit <- model.obj$fit

# 2. data processing

vardrop <- c("m_to_truck","m_to_bus", "m_to_oil","m_to_6oil", "m_to_main_cityhall","m_to_local_cityhall", "satellite_NO2","no2_behr_2005","no2_behr_2006","no2_behr_2007")

desc.vars <- union(desc.vars,

c('pollutant_conc','location_id','native_id','latitude','longitude', 'monitor_type'))

covars.pred <- covar.pred[,!grepl(paste(vardrop,collapse="|"), names(covar.pred))]

covars.pred <- as.data.table(covars.pred)

covars.pred <- log_transform_distances_dt(covars.pred, desc.vars=desc.vars)

covars.pred <- combine_a23_ll_dt(covars.pred, desc.vars=desc.vars)

covars.pred <- as.data.frame(covars.pred)

# 3. pls

if(n.var!=0){

x.covars <- as.matrix(covars.pred[,match(colnames(covars), names(covars.pred))])

x.m <- matrix(rep(apply(covars,2,mean), each=nrow(x.covars)), ncol=ncol(x.covars))

x.sd <- matrix(rep(apply(covars,2,sd), each=nrow(x.covars)), ncol=ncol(x.covars))

x <- (x.covars - x.m)/x.sd

pls.pred <- predict(pls, newdata=x, comps=1:3, type="score")

}

# 4. kriging

coords <- covars.pred[,c("lambert_x","lambert_y")]/1000

numchunk <- floor(nrow(covars.pred)/nchunk)+1

for(i in 1:numchunk){

Start <- (i-1)*nchunk +1

Stop <- min(i*nchunk, nrow(covars.pred))

print(paste(date(), "Predicting for locations", Start, "to", Stop, "of", nrow(covars.pred)))

ind <- Start:Stop

if(n.var!=0){

data.pred <- data.frame(x=coords[ind,1], y=coords[ind,2], pol=rep(1,length(ind)),

comp1=pls.pred[ind,1], comp2=pls.pred[ind,2], comp3=pls.pred[ind,3])

geodata.pred <- as.geodata(data.pred, covar.col=c(4:ncol(data.pred)))

krige <- krige.conv( geodata, loc=data.pred[,c("x","y")],

krige=krige.control(obj.m=fit,

trend.d=trend.spatial(~ comp1+comp2+comp3, geodata),

trend.l=trend.spatial(~ comp1+comp2+comp3, geodata.pred)),

output=output.control(messages=F) )

} else {

data.pred <- data.frame(x=coords[ind,1], y=coords[ind,2], pol=rep(1,length(ind)))

geodata.pred <- as.geodata(data.pred)

krige <- krige.conv( geodata, loc=data.pred[,c("x","y")],

krige=krige.control(obj.m=fit, trend.d="cte", trend.l="cte"),

output=output.control(messages=F) )

}

pred <- krige$predict

unc <- sqrt(krige$krige.var)

if(i==1){

predT <- pred

uncT <- unc

} else {

predT <- c(predT, pred)

uncT <- c(uncT, unc)

}

}

out <- data.frame(native_id=covars.pred$native_id,

longitude=covars.pred$longitude, latitude=covars.pred$latitude,

lambert_x=covars.pred$lambert_x, lambert_y=covars.pred$lambert_y,

pls.pred, pred=predT^2, unc=uncT^2)

# 5. output

return(out)

}
