# Supplementary material for: Concentrations of criteria pollutants in the contiguous U.S., 1979 – 2015: Role of prediction model parsimony in integrated empirical geographic regression
Source: PLoS One. 2020 Feb 18;15(2):e0228535. doi: 10.1371/journal.pone.0228535 (PMC7028280; doi:10.1371/journal.pone.0228535)
Supplement: S1 Table — (DOCX) [file pone.0228535.s002.docx]

Table S1. List of geographical variables and satellite air pollution estimates

| Category | Measure | Variable description^b^ |
| --- | --- | --- |
| Traffic | Distance to the nearest road^a^ | Any road, A1, intersection |
|  | Sum within buffers of 0.05-15 km | A1, A2+A3, truck route, intersections |
| Population | Sum within buffers of 0.5-3 km | Population in block groups |
| Land use/land | Percent within buffers of 0.05-15 km | Urban or Built-Up land |
| cover (Urban) |  | (residential, commercial, industrial, transportation, urban) |
|  |  | Developed low, medium, and high density |
|  |  | Developed open space |
| Land use/land | Percent within buffers of 0.05-15 km | Agricultural land (cropland, groves, feeding) |
| cover (Rural) |  | Rangeland (herbaceous, shrub) |
|  |  | Forest land (deciduous, evergreen, mixed) |
|  |  | Water (streams, lakes, reservoirs, bays) |
|  |  | Wetland |
|  |  | Barren land (beaches, dry salt flats, sand, mines, rock) |
|  |  | Tundra |
|  |  | Perennial snow or Ice |
| Position | Coordinates | Longitude, latitude |
| Source | Distance to the nearest source^a^ | Coastline |
|  |  | Commercial area |
|  |  | Railroad |
|  |  | Railyard |
|  |  | Airport |
|  |  | Major airport |
|  |  | Large port |
| Emissions | Sum of site-specific facility emissions | PM_2.5_ |
|  | within buffers of 3-30 km | PM_10_ |
|  |  | CO |
|  |  | SO_2_ |
|  |  | NO_X_ |
| Vegetation | Quantiles within buffers of 0.5-10 km | Normalized Difference Vegetation Index (NDVI) |
| Imperviousness | Percent within buffers of 0.05-5 km | Impervious surface value |
| Elevation | Elevation above sea levels | Elevation value |
|  | Counts of points above or below a threshold within buffers of 1-5 km |  |
| Satellite-based |  | PM_2.5_ |
| air pollution |  | NO_2_ |
| estimates |  | CO |
|  |  | SO_2_ |
|  |  | HCHO |

a. Distances calculated to spatial features are truncated at 25 km

b. See the Multi-Ethnic Study of Atherosclerosis and Air pollution (MESA Air) Data Organization and Operating Procedures (DOOP) for data sources for these variables (https://www.uwchscc.org/MESAAP/Documents/MESAAirDOOP.pdf).
