# Supplementary material for: Concentrations of criteria pollutants in the contiguous U.S., 1979 – 2015: Role of prediction model parsimony in integrated empirical geographic regression
Source: PLoS One. 2020 Feb 18;15(2):e0228535. doi: 10.1371/journal.pone.0228535 (PMC7028280; doi:10.1371/journal.pone.0228535)
Supplement: S2 Table — (DOCX) [file pone.0228535.s003.docx]

Table S2. Available years of satellite estimates for air pollution and metrics used for national prediction models

| Pollutant | Available years | Metrics for years with data | Metrics for years without data |
| --- | --- | --- | --- |
| NO_2_ | 2005-2015 | Annual average | 3-year average for the earliest or latest 3 years ^a^ |
|  |  | 3-year average | 3-year averages for the earliest or latest 3 years  3-year average for the earliest or latest 3 years |
| SO_2_ | 2005-2016 | Annual average | 3-year average for the earliest or latest 3 years |
| CO | 2001-2016 | Annual average | 3-year average for the earliest or latest 3 years |
| HCHO | 2005-2016 | 12-year average | 12-year average |
| PM_2.5_ | 1998-2014 | Annual average | 3-year average for the earliest or latest 3 years  3-year average for the earliest or latest 3 years |

a. 3-year average for 2005-2007 before 2005
