# Supplementary material for: Concentrations of criteria pollutants in the contiguous U.S., 1979 – 2015: Role of prediction model parsimony in integrated empirical geographic regression
Source: PLoS One. 2020 Feb 18;15(2):e0228535. doi: 10.1371/journal.pone.0228535 (PMC7028280; doi:10.1371/journal.pone.0228535)
Supplement: S3 Table — (DOCX) [file pone.0228535.s004.docx]

Table S3. Summary statistics of annual average concentrations for six criteria air pollutants across regulatory monitoring sites in the contiguous U.S. for 1980, 1990, 2000, and 2010

| Pollutant | Year | N | Percentile | | | | | Mean | SD |
| --- | --- | --- | --- | --- | --- | --- | --- | --- | --- |
|  |  |  | 10 | 25 | 50 | 75 | 90 |  |  |
| NO_2_ | 1980 | 138 | 8.4 | 16.9 | 24.8 | 34.4 | 49.8 | 26.5 | 15.2 |
| (ppb) | 1990 | 266 | 6.0 | 11.4 | 17.8 | 24.9 | 31.8 | 18.9 | 10.5 |
|  | 2000 | 345 | 5.4 | 9.7 | 15.5 | 20.3 | 26.2 | 15.6 | 8.2 |
|  | 2010 | 327 | 2.8 | 5.2 | 9.1 | 13.1 | 17.3 | 9.6 | 5.6 |
| SO_2_ | 1980 | 552 | 3.3 | 6.5 | 10.5 | 15.6 | 23.9 | 12.7 | 10.4 |
| (ppb) | 1990 | 619 | 1.6 | 3.6 | 7.1 | 9.7 | 13.5 | 7.3 | 4.8 |
|  | 2000 | 496 | 1.4 | 2.4 | 4.2 | 6.2 | 9.0 | 4.7 | 2.9 |
|  | 2010 | 370 | 1.0 | 1.1 | 1.6 | 2.8 | 4.2 | 2.2 | 1.6 |
| Ozone | 1980 | 288 | 37.8 | 45.8 | 52.0 | 59.4 | 66.2 | 52.0 | 11.3 |
| (ppb) | 1990 | 492 | 39.3 | 44.9 | 49.3 | 54.1 | 59.4 | 49.3 | 7.8 |
|  | 2000 | 768 | 39.6 | 44.7 | 50.1 | 54.8 | 58.4 | 49.4 | 7.4 |
|  | 2010 | 850 | 37.2 | 41.6 | 46.6 | 51.0 | 53.7 | 45.8 | 6.8 |
| CO | 1990 | 277 | 0.48 | 0.66 | 0.95 | 1.26 | 1.67 | 1.02 | 0.48 |
| (ppm) | 2000 | 293 | 0.33 | 0.41 | 0.54 | 0.76 | 0.99 | 0.62 | 0.28 |
|  | 2010 | 218 | 0.29 | 0.31 | 0.33 | 0.38 | 0.46 | 0.35 | 0.10 |
| PM_10_ | 1990 | 946 | 18.6 | 23.1 | 27.6 | 33.6 | 39.6 | 29.0 | 10.0 |
| (μg/m^3^) | 2000 | 1,021 | 12.9 | 18.1 | 22.7 | 27.2 | 35.3 | 23.8 | 10.3 |
|  | 2010 | 829 | 8.8 | 13.6 | 18.0 | 22.4 | 27.9 | 18.6 | 8.3 |
| PM_2.5_ | 2000 | 950 | 6.8 | 10.1 | 12.8 | 15.5 | 17.1 | 12.5 | 4.1 |
| (μg/m^3^) | 2010 | 934 | 4.4 | 7.2 | 9.5 | 11.3 | 12.5 | 9.0 | 3.0 |
