# Supplementary material for: Concentrations of criteria pollutants in the contiguous U.S., 1979 – 2015: Role of prediction model parsimony in integrated empirical geographic regression
Source: PLoS One. 2020 Feb 18;15(2):e0228535. doi: 10.1371/journal.pone.0228535 (PMC7028280; doi:10.1371/journal.pone.0228535)
Supplement: S4 Table — (DOCX) [file pone.0228535.s005.docx]

Table S4. Medians of estimated covariance parameters and proportions of predictions explained by PLS regression across the Integrated Empirical Geographic (IEG) models including different numbers of variables for NO_2_ and PM_2.5_ in 2000

|  |  |  |  | Number of variables | | | | |
| --- | --- | --- | --- | --- | --- | --- | --- | --- |
| Pollutant | CV |  |  | 0 | 5 | 10 | 30 | All |
| NO_2_ | Conventional | Covariance | Range (km) | 79.99 | 192.07 | 177.26 | 207.02 | 179.52 |
|  |  | parameter | Partial sill | 0.91 | 0.13 | 0.10 | 0.11 | 0.17 |
|  |  |  | Nugget | 0.12 | 0.10 | 0.10 | 0.08 | 0.09 |
|  |  | Prediction | Ratio^a^ | 0.79 | 0.96 | 0.96 | 0.94 | 0.92 |
|  | Clustered | Covariance | Range (km) | 74.33 | 198.27 | 197.70 | 176.70 | 250.00 |
|  |  | parameter | Partial sill | 0.90 | 0.13 | 0.10 | 0.08 | 0.14 |
|  |  |  | Nugget | 0.11 | 0.10 | 0.09 | 0.08 | 0.10 |
|  |  | Prediction | Ratio^a^ | 1.00 | 0.99 | 1.00 | 1.00 | 0.98 |
| PM_2.5_ | Conventional | Covariance | Range (km) | 255.05 | 234.61 | 97.06 | 162.13 | 283.35 |
|  |  | parameter | Partial sill | 0.40 | 0.11 | 0.08 | 0.09 | 0.16 |
|  |  |  | Nugget | 0.02 | 0.03 | 0.02 | 0.02 | 0.02 |
|  |  | Prediction | Ratio^a^ | 0.73 | 0.93 | 0.98 | 0.95 | 0.88 |
|  | Clustered | Covariance | Range (km) | 241.10 | 199.10 | 107.43 | 154.03 | 230.46 |
|  |  | parameter | Partial sill | 0.42 | 0.12 | 0.09 | 0.09 | 0.14 |
|  |  |  | Nugget | 0.02 | 0.03 | 0.02 | 0.02 | 0.02 |
|  |  | Prediction | Ratio^a^ | 0.88 | 0.97 | 1.00 | 0.99 | 0.96 |

a. Proportion of predictions by PLS predictors to prediction by PLS predictors and kriging
