# Supplementary material for: Concentrations of criteria pollutants in the contiguous U.S., 1979 – 2015: Role of prediction model parsimony in integrated empirical geographic regression
Source: PLoS One. 2020 Feb 18;15(2):e0228535. doi: 10.1371/journal.pone.0228535 (PMC7028280; doi:10.1371/journal.pone.0228535)
Supplement: S5 Table — (DOCX) [file pone.0228535.s006.docx]

Table S5. CV statistics of IEG models for annual average concentrations of NO_2_ and PM_2.5_ in 2000 using PLS predictors estimated from subsets of geographic variables chosen by forward selection and least absolute shrinkage and selection operator (lasso)

|  |  |  | RMSE | | | | | | R^2^ | | | | | |
| --- | --- | --- | --- | --- | --- | --- | --- | --- | --- | --- | --- | --- | --- | --- |
| N of variables | | | 3 | 10 | 20 | 30 | 60 | 120 | 3 | 10 | 20 | 30 | 60 | 120 |
| Pollutant | CV | Selection method |  |  |  |  |  |  |  |  |  |  |  |  |
| NO_2_ | Conventional | Forward | 0.21 | 0.19 | 0.18 | 0.18 | 0.21 | 0.21 | 0.84 | 0.87 | 0.88 | 0.88 | 0.85 | 0.85 |
|  |  | Lasso | 0.21 | 0.19 | 0.17 | 0.18 | 0.19 | 0.22 | 0.84 | 0.87 | 0.89 | 0.88 | 0.87 | 0.83 |
|  | Clustered | Forward | 0.27 | 0.23 | 0.22 | 0.23 | 0.26 | 0.27 | 0.74 | 0.81 | 0.83 | 0.82 | 0.76 | 0.73 |
|  |  | Lasso | 0.29 | 0.26 | 0.25 | 0.26 | 0.25 | 0.31 | 0.69 | 0.76 | 0.78 | 0.77 | 0.77 | 0.65 |
| PM_2.5_ | Conventional | Forward | 0.13 | 0.13 | 0.12 | 0.12 | 0.12 | 0.13 | 0.84 | 0.85 | 0.86 | 0.86 | 0.86 | 0.84 |
|  |  | Lasso | 0.15 | 0.12 | 0.11 | 0.11 | 0.11 | 0.12 | 0.79 | 0.87 | 0.88 | 0.88 | 0.88 | 0.88 |
|  | Clustered | Forward | 0.23 | 0.21 | 0.20 | 0.21 | 0.21 | 0.22 | 0.50 | 0.58 | 0.62 | 0.59 | 0.58 | 0.55 |
|  |  | Lasso | 0.25 | 0.21 | 0.21 | 0.20 | 0.20 | 0.21 | 0.43 | 0.60 | 0.60 | 0.62 | 0.64 | 0.58 |
