# Supplementary material for: Concentrations of criteria pollutants in the contiguous U.S., 1979 – 2015: Role of prediction model parsimony in integrated empirical geographic regression
Source: PLoS One. 2020 Feb 18;15(2):e0228535. doi: 10.1371/journal.pone.0228535 (PMC7028280; doi:10.1371/journal.pone.0228535)
Supplement: S6 Table — (DOCX) [file pone.0228535.s007.docx]

Table S6. Cross-validation (CV) statistics of the ozone national Integrated Empirical Geographic (IEG) models including no some and all geographic variables and/or satellite estimates for four metrics by summer and all seasons

|  |  |  |  |  | Conventional CV | | | Clustered CV | | |
| --- | --- | --- | --- | --- | --- | --- | --- | --- | --- | --- |
| Season | Metric | N of sites | Year | N of variables | Median | 25% | 75% | Median | 25% | 75% |
| All | 24-hr mean | 213-479 | 1979-1986 | 0 | 0.38 | 0.26 | 0.47 | 0.00 | 0.00 | 0.00 |
|  |  |  | 1990-2015 | 3-30^a^ | 0.73 | 0.68 | 0.75 | 0.63 | 0.55 | 0.66 |
|  |  |  |  | All | 0.64 | 0.59 | 0.67 | 0.40 | 0.25 | 0.50 |
|  | 8-hr max |  |  | 0 | 0.57 | 0.45 | 0.65 | 0.14 | 0.03 | 0.19 |
|  |  |  |  | 3-30 | 0.70 | 0.64 | 0.74 | 0.46 | 0.32 | 0.54 |
|  |  |  |  | All | 0.67 | 0.59 | 0.71 | 0.25 | 0.11 | 0.40 |
|  | 8-hr mean |  |  | 0 | 0.60 | 0.48 | 0.67 | 0.17 | 0.07 | 0.23 |
|  |  |  |  | 3-30 | 0.71 | 0.66 | 0.77 | 0.50 | 0.36 | 0.57 |
|  |  |  |  | All | 0.69 | 0.61 | 0.74 | 0.32 | 0.16 | 0.45 |
|  | 1-hr max |  | 1980-1986 | 0 | 0.57 | 0.44 | 0.63 | 0.18 | 0.06 | 0.29 |
|  |  |  | 1990-2015 | 3-30 | 0.67 | 0.58 | 0.73 | 0.43 | 0.23 | 0.50 |
|  |  |  |  | All | 0.63 | 0.52 | 0.69 | 0.18 | 0.00 | 0.35 |
| Summer | 24-hr mean | 232-916 | 1979-2015 | 0 | 0.50 | 0.46 | 0.57 | 0.08 | 0.00 | 0.14 |
| (May-Sep) |  |  |  | 3-30 | 0.69 | 0.62 | 0.73 | 0.46 | 0.29 | 0.52 |
|  |  |  |  | All | 0.61 | 0.55 | 0.64 | 0.28 | 0.14 | 0.36 |
|  | 8-hr max |  |  | 0 | 0.72 | 0.65 | 0.77 | 0.34 | 0.17 | 0.46 |
|  |  |  |  | 3-30 | 0.73 | 0.68 | 0.78 | 0.41 | 0.24 | 0.52 |
|  |  |  |  | All | 0.73 | 0.65 | 0.78 | 0.36 | 0.16 | 0.47 |
|  | 8-hr mean |  |  | 0 | 0.72 | 0.65 | 0.78 | 0.32 | 0.16 | 0.47 |
|  |  |  |  | 3-30 | 0.74 | 0.66 | 0.79 | 0.41 | 0.23 | 0.52 |
|  |  |  |  | All | 0.73 | 0.64 | 0.79 | 0.36 | 0.15 | 0.48 |
|  | 1-hr max |  |  | 0 | 0.73 | 0.57 | 0.77 | 0.36 | 0.22 | 0.47 |
|  |  |  |  | 3-30 | 0.74 | 0.59 | 0.78 | 0.39 | 0.27 | 0.54 |
|  |  |  |  | All | 0.74 | 0.58 | 0.78 | 0.32 | 0.18 | 0.48 |

a. Summaries of the highest R^2^s among years
