# Supplementary material for: Concentrations of criteria pollutants in the contiguous U.S., 1979 – 2015: Role of prediction model parsimony in integrated empirical geographic regression
Source: PLoS One. 2020 Feb 18;15(2):e0228535. doi: 10.1371/journal.pone.0228535 (PMC7028280; doi:10.1371/journal.pone.0228535)
Supplement: S2 Fig — (DOCX) [file pone.0228535.s009.docx]

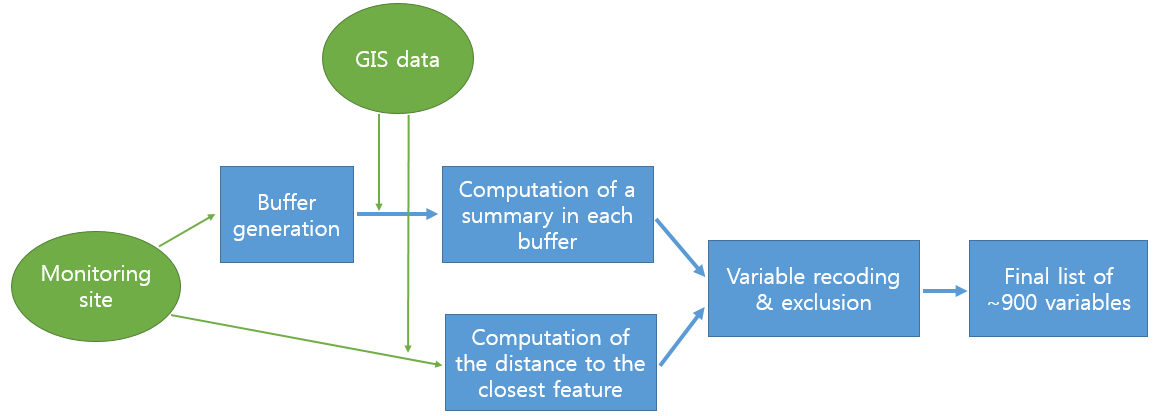


F2. Fig. Diagram of data preprocessing and variable computation for more than 900 geographic variables.
