# Supplementary material for: Concentrations of criteria pollutants in the contiguous U.S., 1979 – 2015: Role of prediction model parsimony in integrated empirical geographic regression
Source: PLoS One. 2020 Feb 18;15(2):e0228535. doi: 10.1371/journal.pone.0228535 (PMC7028280; doi:10.1371/journal.pone.0228535)
Supplement: S4 Fig — (DOCX) [file pone.0228535.s011.docx]

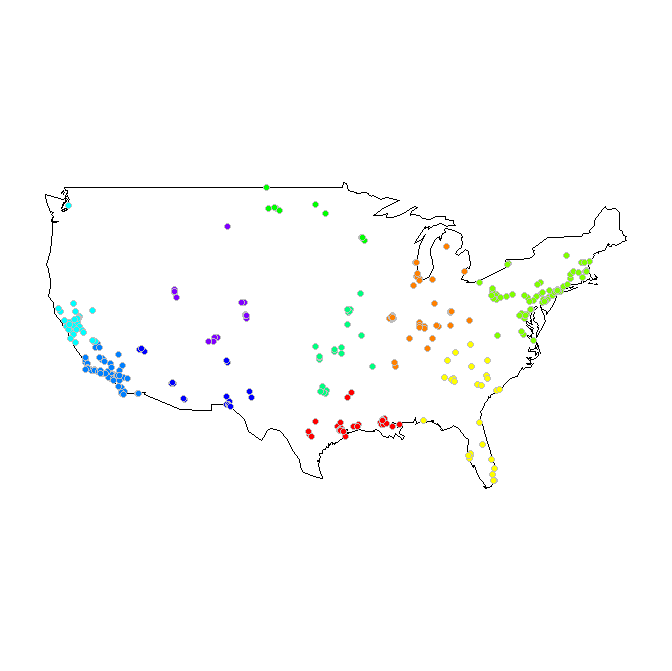
 Figure S4. Map of 10 spatial clusters of 345 NO_2_ regulatory monitoring sites in 2000 determined by k-means clustering
