# Supplementary material for: Concentrations of criteria pollutants in the contiguous U.S., 1979 – 2015: Role of prediction model parsimony in integrated empirical geographic regression
Source: PLoS One. 2020 Feb 18;15(2):e0228535. doi: 10.1371/journal.pone.0228535 (PMC7028280; doi:10.1371/journal.pone.0228535)
Supplement: S5 Fig — (DOCX) [file pone.0228535.s012.docx]

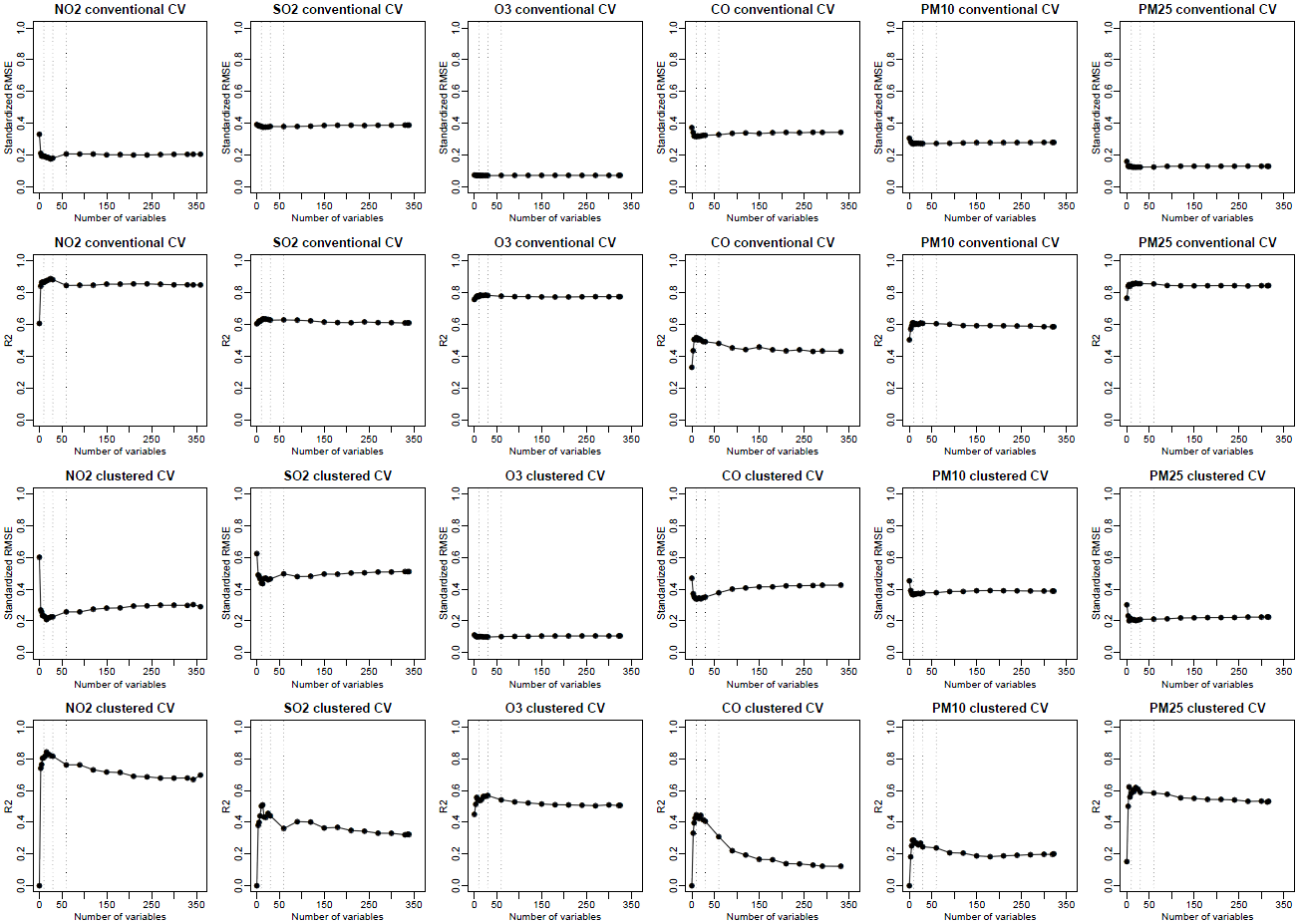


Figure S5. The relationship between numbers of variables and cross-validation (CV) statistics from the national Integrated Empirical Geographic (IEG) models of six criteria air pollutants in the year 2000 by conventional and clustered cross-validation (number of variables on the x-axis of plots; standardized MSE on the y-axis of plots for the first and third rows and R^2^ for the second and fourth rows; vertical lines for 10, 30, and 60)
