# Supplementary material for: Concentrations of criteria pollutants in the contiguous U.S., 1979 – 2015: Role of prediction model parsimony in integrated empirical geographic regression
Source: PLoS One. 2020 Feb 18;15(2):e0228535. doi: 10.1371/journal.pone.0228535 (PMC7028280; doi:10.1371/journal.pone.0228535)
Supplement: S6 Fig — (DOCX) [file pone.0228535.s013.docx]

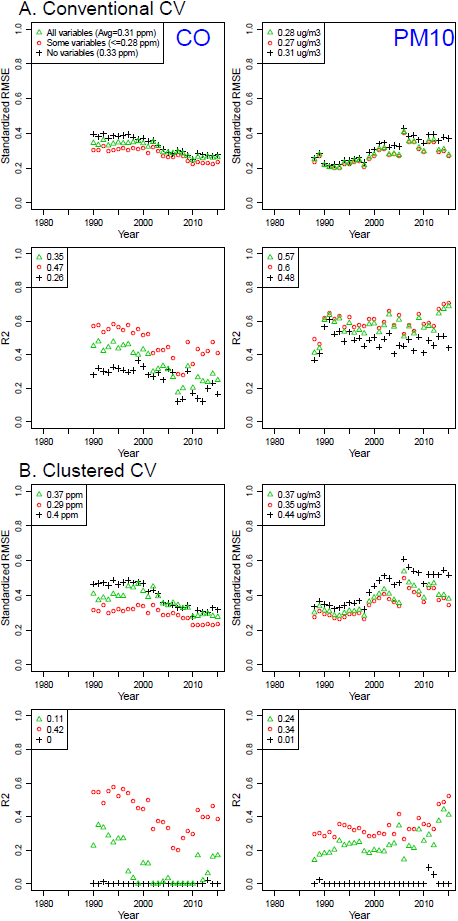


Figure S6. Standardized RMSEs and R^2^s of the national Integrated Empirical Geographic (IEG) models including no, some, and all variables from conventional and clustered cross-validation (CV) during 1979-2015 for the contiguous U.S. by CO and PM_10_
