# Supplementary material for: Concentrations of criteria pollutants in the contiguous U.S., 1979 – 2015: Role of prediction model parsimony in integrated empirical geographic regression
Source: PLoS One. 2020 Feb 18;15(2):e0228535. doi: 10.1371/journal.pone.0228535 (PMC7028280; doi:10.1371/journal.pone.0228535)
Supplement: S7 Fig — For ease of reading, figures include horizontal lines for y-axis values of 30, 50, and 100. (DOCX) [file pone.0228535.s014.docx]

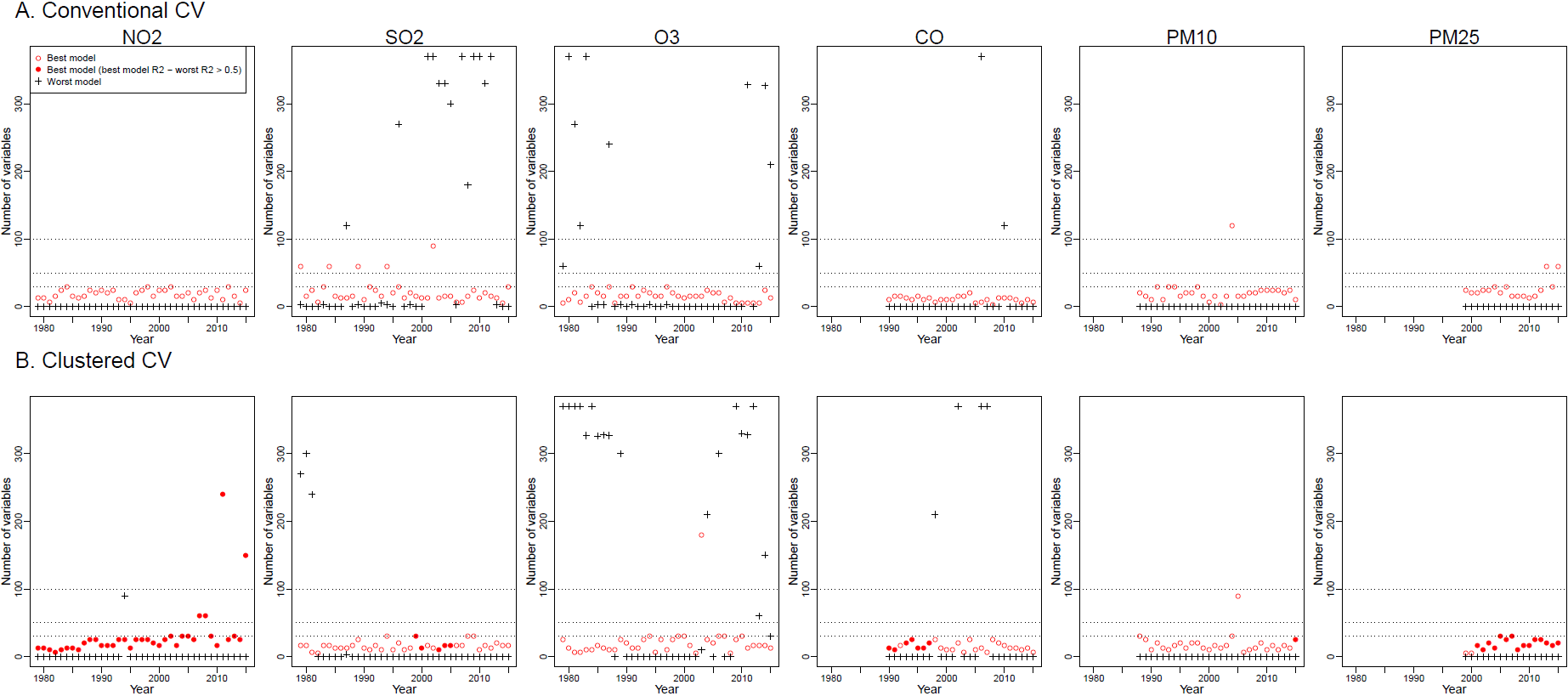


Figure S7. Numbers of variables selected for the best and worst national Integrated Empirical Geographic (IEG) models with the highest and lowest cross-validated R^2^s, respectively (which was also the model with the lowest and highest standardized root mean square error), by pollutant and CV type (conventional CV, clustered CV). For ease of reading, figures include horizontal lines for y-axis values of 30, 50, and 100.
