# Supplementary material for: Concentrations of criteria pollutants in the contiguous U.S., 1979 – 2015: Role of prediction model parsimony in integrated empirical geographic regression
Source: PLoS One. 2020 Feb 18;15(2):e0228535. doi: 10.1371/journal.pone.0228535 (PMC7028280; doi:10.1371/journal.pone.0228535)
Supplement: S8 Fig — (DOCX) [file pone.0228535.s015.docx]

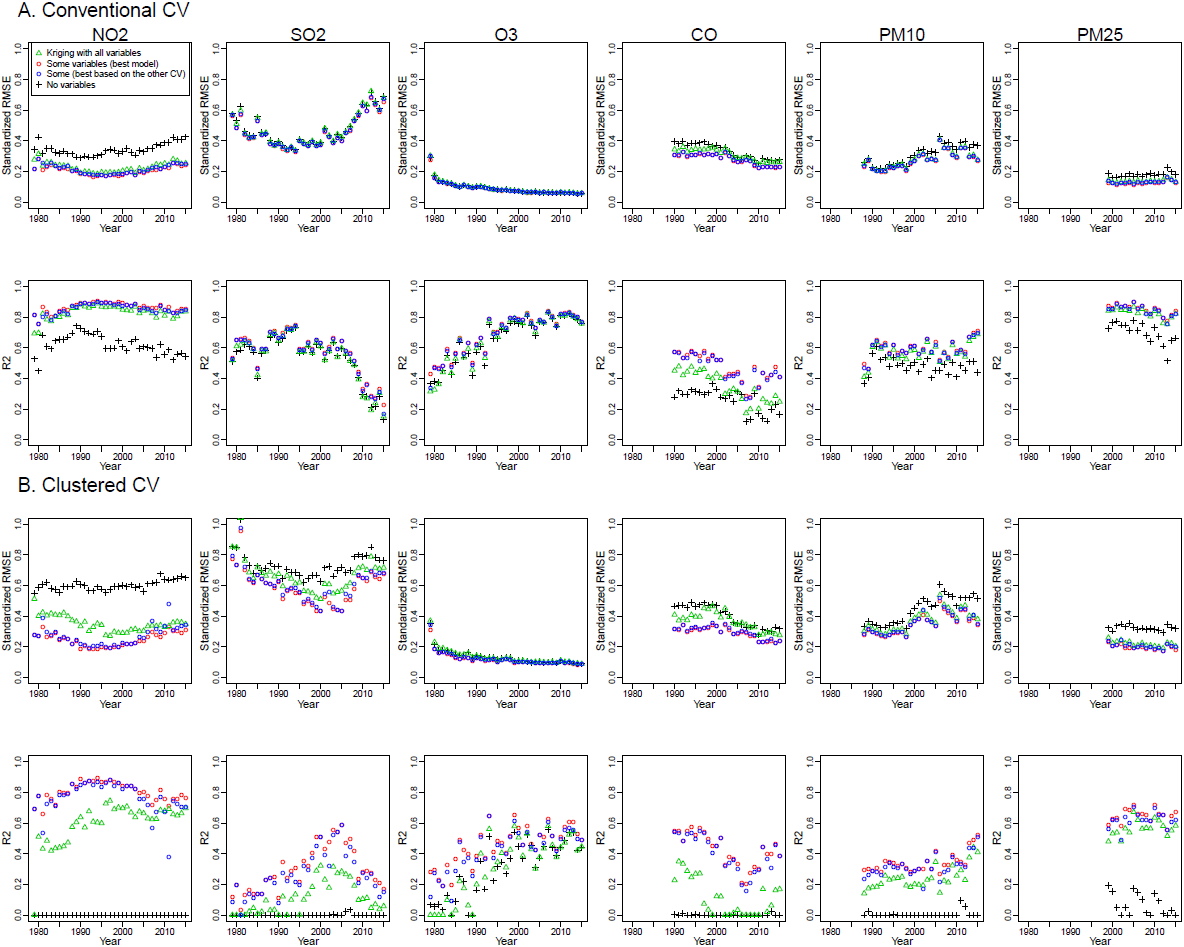


Figure S8. Standardized root mean square errors and R^2^s of the national Integrated Empirical Geographic (IEG) models including no variables, some variables (i.e., between 3 and 30 variables), and all variables from conventional and clustered cross-validation, by year and pollutant, for the contiguous U.S.
