# Supplementary material for: Concentrations of criteria pollutants in the contiguous U.S., 1979 – 2015: Role of prediction model parsimony in integrated empirical geographic regression
Source: PLoS One. 2020 Feb 18;15(2):e0228535. doi: 10.1371/journal.pone.0228535 (PMC7028280; doi:10.1371/journal.pone.0228535)
Supplement: S9 Fig — (DOCX) [file pone.0228535.s016.docx]

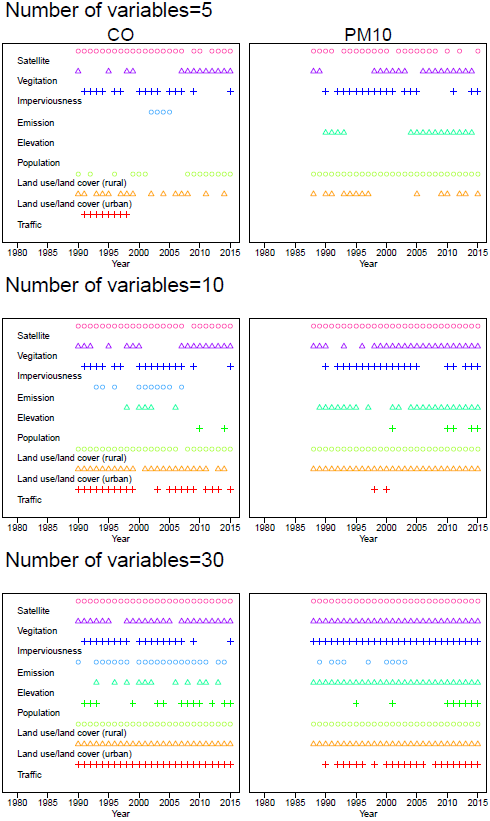


Figure S9. Categories (nine out of the eleven in S1 Table) of geographic variables chosen by forward selection for the national Integrated Empirical Geographic (IEG) models by year, pollutant (CO and PM10), and number of variables (5, 10, and 30) during 1979-2015 for the contiguous U.S.
