# Supplementary material for: Concentrations of criteria pollutants in the contiguous U.S., 1979 – 2015: Role of prediction model parsimony in integrated empirical geographic regression
Source: PLoS One. 2020 Feb 18;15(2):e0228535. doi: 10.1371/journal.pone.0228535 (PMC7028280; doi:10.1371/journal.pone.0228535)
Supplement: S10 Fig — For ease of reading, vertical lines are shown at x-axis values of 10, 30, and 60. (DOCX) [file pone.0228535.s017.docx]

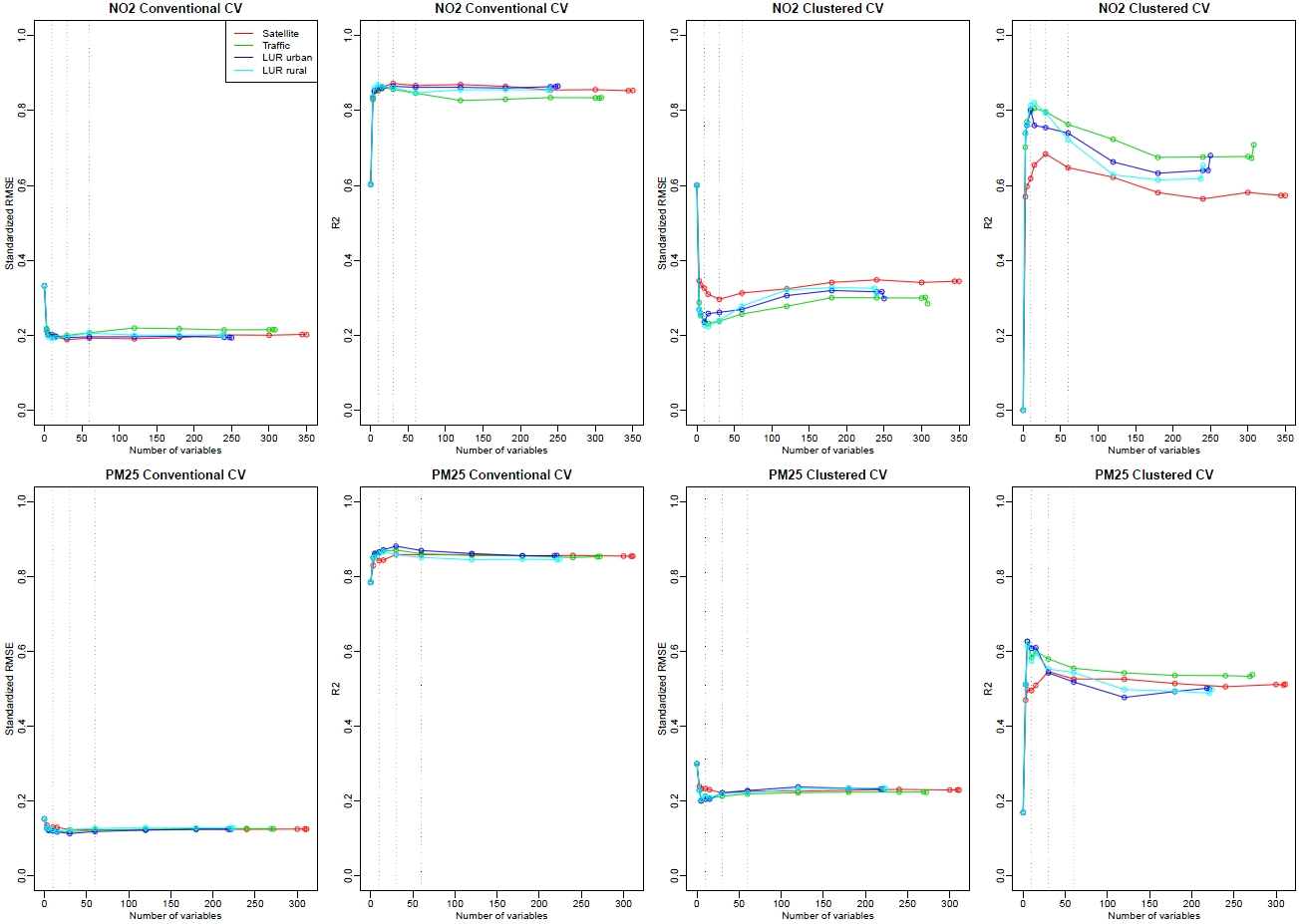


Figure S10. The relationship between numbers of variables and cross-validation (CV) statistics from the national Integrated Empirical Geographic (IEG) models of NO_2_ and PM_2.5_ in 2000 by exclusion of a different category of geographic variables and satellite air pollution estimates by conventional and clustered cross-validation. For ease of reading, vertical lines are shown at x-axis values of 10, 30, and 60.
