# Supplementary material for: Concentrations of criteria pollutants in the contiguous U.S., 1979 – 2015: Role of prediction model parsimony in integrated empirical geographic regression
Source: PLoS One. 2020 Feb 18;15(2):e0228535. doi: 10.1371/journal.pone.0228535 (PMC7028280; doi:10.1371/journal.pone.0228535)
Supplement: S11 Fig — For easy of viewing, vertical lines are shown at x-axis values of 10, 30, and 60. (DOCX) [file pone.0228535.s018.docx]

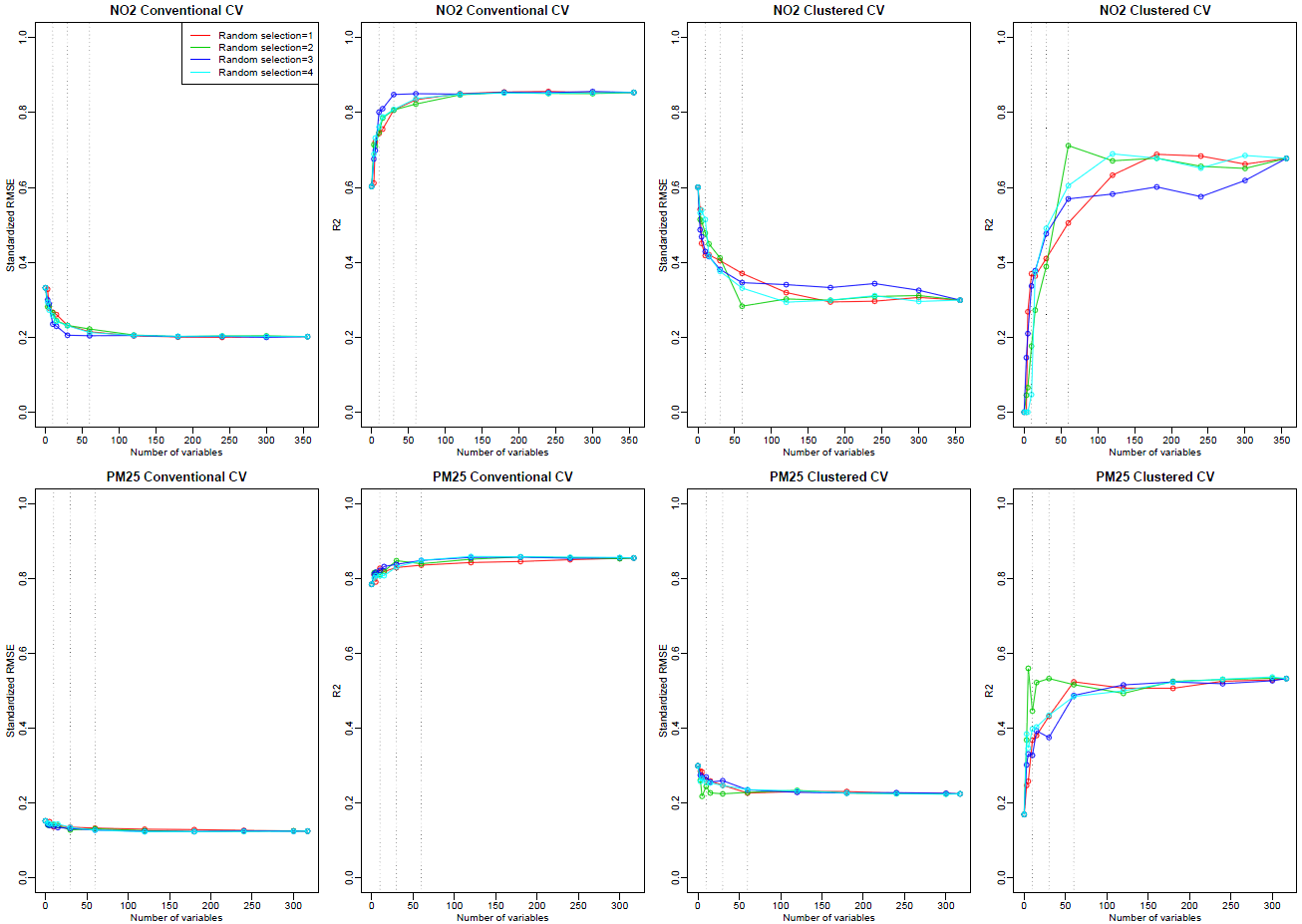


Figure S11. The relationship between the same numbers of randomly selected variables as those in forward selection and cross-validation (CV) statistics from the national Integrated Empirical Geographic (IEG) models of NO_2_ and PM_2.5_ in 2000 by conventional and clustered cross-validation. For easy of viewing, vertical lines are shown at x-axis values of 10, 30, and 60
