# Supplementary material for: Concentrations of criteria pollutants in the contiguous U.S., 1979 – 2015: Role of prediction model parsimony in integrated empirical geographic regression
Source: PLoS One. 2020 Feb 18;15(2):e0228535. doi: 10.1371/journal.pone.0228535 (PMC7028280; doi:10.1371/journal.pone.0228535)
Supplement: S12 Fig — Vertical lines shown for x-axis values of 10, 30, and 60. (DOCX) [file pone.0228535.s019.docx]

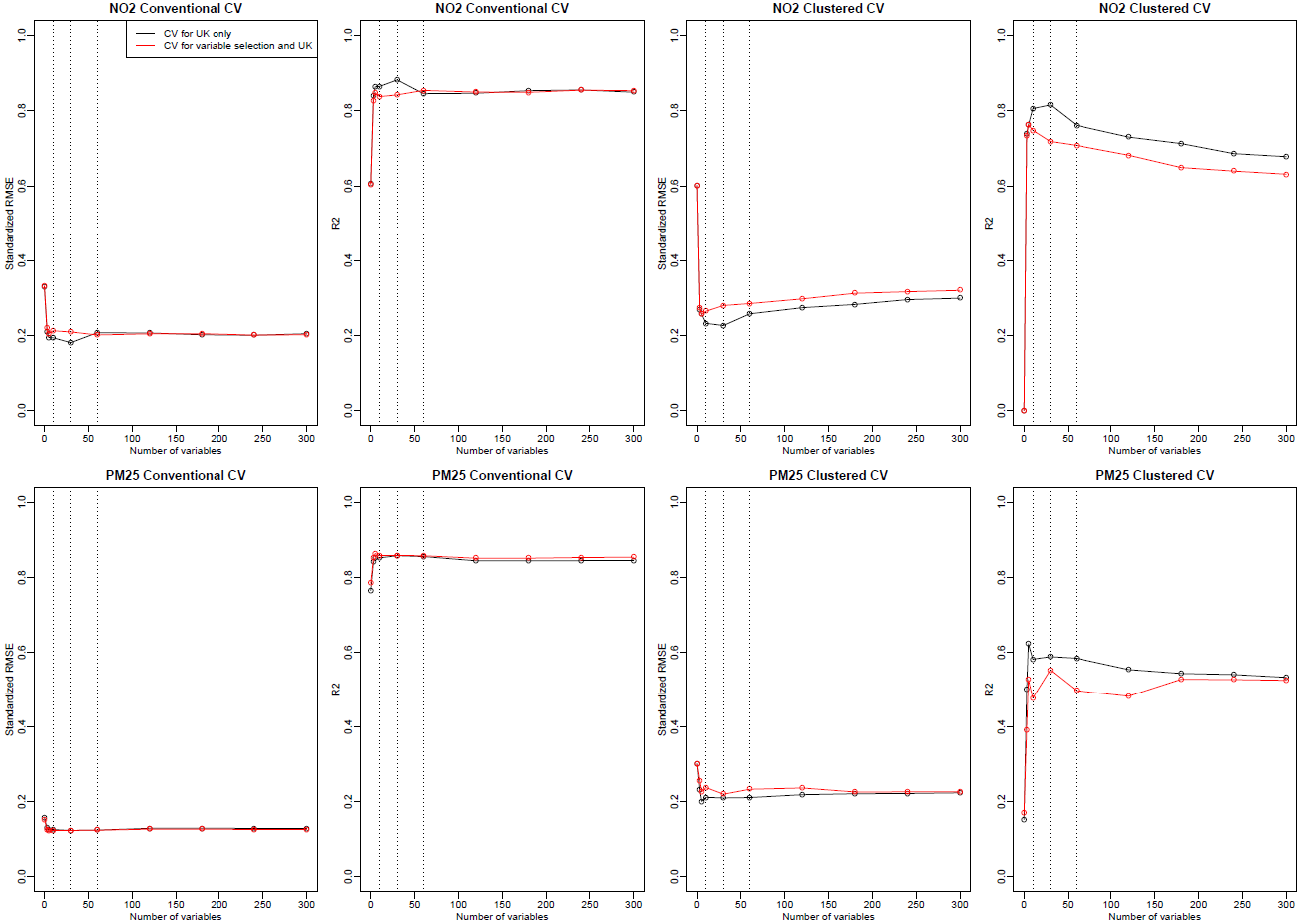
Figure S12. The relationship between numbers of variables and cross-validation (CV) statistics to compare including forward selection and estimation of PLS predictors, with parameter estimation alone in the national Integrated Empirical Geographic (IEG) models of NO_2_ and PM_2.5_ in 2000 by conventional and clustered CV. Vertical lines shown for x-axis values of 10, 30, and 60.
