# Supplementary material for: Concentrations of criteria pollutants in the contiguous U.S., 1979 – 2015: Role of prediction model parsimony in integrated empirical geographic regression
Source: PLoS One. 2020 Feb 18;15(2):e0228535. doi: 10.1371/journal.pone.0228535 (PMC7028280; doi:10.1371/journal.pone.0228535)
Supplement: S14 Fig — (DOCX) [file pone.0228535.s021.docx]

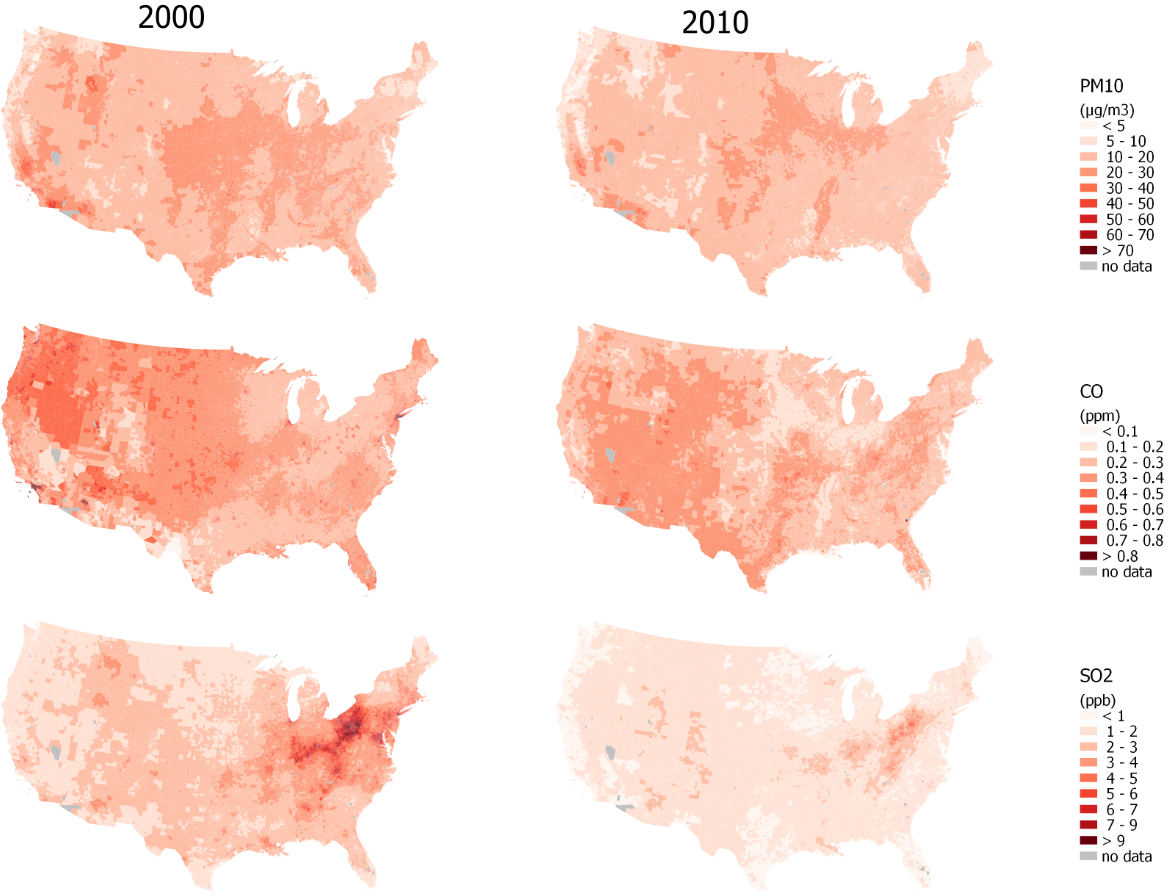


Figure S14. Maps of Census Block Group population-weighted mean for PM_10_, CO, and SO_2_ from the “best” Integrated Empirical Geographic (IEG) models mostly including 3-30 geographic variables for 2000 and 2010 in the contiguous U.S.
