# Supplementary material for: Concentrations of criteria pollutants in the contiguous U.S., 1979 – 2015: Role of prediction model parsimony in integrated empirical geographic regression
Source: PLoS One. 2020 Feb 18;15(2):e0228535. doi: 10.1371/journal.pone.0228535 (PMC7028280; doi:10.1371/journal.pone.0228535)
Supplement: S15 Fig — (DOCX) [file pone.0228535.s022.docx]

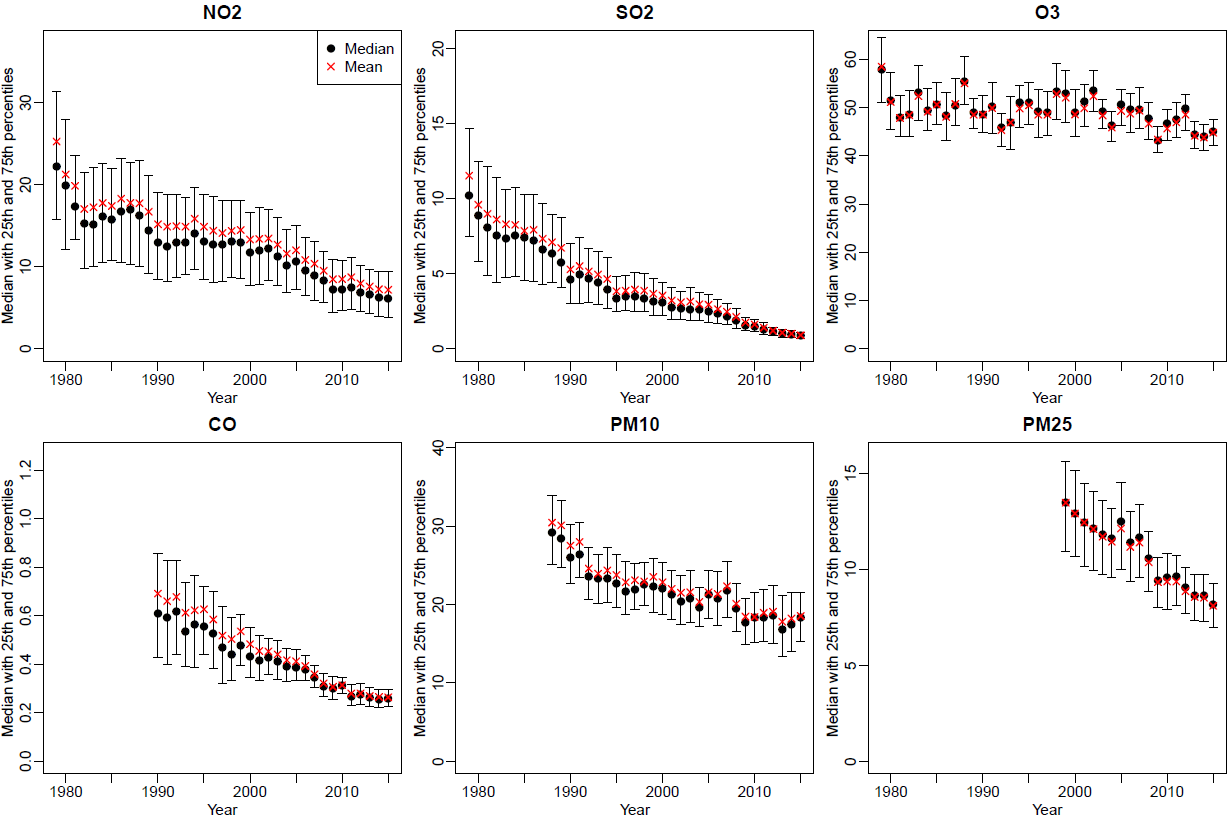
Figure S15. Quantile-based plots of population-weighted annual average concentrations of six criteria air pollutants across 215,491 Census Block Group centroids, based on predicted concentrations at Census Block centroids by using the “best” Integrated Empirical Geographic (IEG) models mostly using 3-30 geographic variables for 1979-2015 in the contiguous U.S.
